# Differential Expression of the Insulin-Like Growth Factor Receptor among Early Breast Cancer Subtypes

**DOI:** 10.1371/journal.pone.0091407

**Published:** 2014-03-17

**Authors:** Giannis Mountzios, Dimitra Aivazi, Ioannis Kostopoulos, Helen P. Kourea, George Kouvatseas, Eleni Timotheadou, Pantelis Zebekakis, Ioannis Efstratiou, Helen Gogas, Chrisanthi Vamvouka, Sofia Chrisafi, Anastasios Stofas, George Pentheroudakis, Angelos Koutras, Eleni Galani, Dimitrios Bafaloukos, George Fountzilas

**Affiliations:** 1 Department of Medical Oncology, 251 Airforce General Hospital, Athens, Greece; 2 Laboratory of Molecular Oncology, Hellenic Foundation for Cancer Research, Aristotle University of Thessaloniki School of Medicine, Thessaloniki, Greece; 3 Department of Pathology, Aristotle University of Thessaloniki School of Medicine, Thessaloniki, Greece; 4 Department of Pathology, University Hospital of Patras, Rion, Greece; 5 Health Data Specialists Ltd, Athens, Greece; 6 Department of Medical Oncology, “Papageorgiou” Hospital, Aristotle University of Thessaloniki School of Medicine, Thessaloniki, Greece; 7 1^st^ Department of Internal Medicine, AHEPA Hospital, Aristotle University of Thessaloniki School of Medicine, Thessaloniki, Greece; 8 Department of Pathology, “Papageorgiou” Hospital, Thessaloniki, Greece; 9 First Department of Medicine, “Laiko” General Hospital, University of Athens, Medical School, Athens, Greece; 10 Department of Pathology, “Evangelismos” Hospital, Athens, Greece; 11 Pathology Department National & Kapodistrian University of Athens, Athens, Greece; 12 Department of Medical Oncology, Ioannina University Hospital, Ioannina, Greece; 13 Division of Oncology, Department of Medicine, University Hospital, University of Patras Medical School, Patras, Greece; 14 Second Department of Medical Oncology, “Metropolitan” Hospital, Piraeus, Greece; 15 First Department of Medical Oncology, “Metropolitan” Hospital, Piraeus, Greece; University of Central Florida, United States of America

## Abstract

**Introduction:**

We sought to determine the level of protein expression of the critical components of the insulin-like growth factor receptor (IGFR) pathway and to evaluate their prognostic significance across the different early breast cancer subtypes.

**Patients and Methods:**

Archival tumor tissue from 1,021 women with early, node positive breast cancer, who were prospectively evaluated within two randomized clinical trials, was used to construct tissue microarrays that were stained for hormone receptors (HR), Ki67, HER2, epidermal growth factor receptor (EGFR) and cytokeratins 5/6, to classify tumors into five immunophenotypical subgroups. Immunohistochemical (IHC) expression of IGF1R-alpha and beta subunits, IGF2R and IGF-binding protein 2 (IGFBP2) was assessed using the immunoreactive score (IRS). Repeated internal cross-validation was performed to examine the statistical validity of the cut off points for all biomarkers.

**Results:**

After a median follow-up time of 105.4 months, overall 370 women (36.2%) had relapsed and 270 (26.4%) had died. Tumors expressing IGF1R-alpha above the median IRS were significantly more frequently HR positive (luminal A+B+HER2), as compared to HER2-enriched and triple negative ones (p<0.001 for both comparisons). IGF2R was overexpressed significantly more frequently in HR negative tumors (p = 0.001) and had an inverse correlation with all other biomarkers. Patients with luminal A and B tumors with high IGF1R-alpha and negative EGFR expression (N = 190) had significantly higher 4-year survival rates, as compared to the rest (log-rank p = 0.046), as did patients with luminal A and B tumors with high IGF1R-alpha and low IGF2R expression, as compared to the rest (N = 91), (log-rank p = 0.035). After adjustment for significant variables, patients in the latter group had a relative 45% reduction in the risk of death, as compared to the rest (p = 0.035).

**Conclusion:**

Aberrant expression of components of the IGF1R pathway is associated with better clinical outcomes in women with luminal A and B, node positive, early breast cancer.

## Introduction

Despite recent advances in molecular biology and therapeutics, breast cancer remains a highly lethal malignancy worldwide [1]. Early breast cancer represents a heterogeneous disease entity, that can be further categorized by the use of simple immunohistochemical (IHC) molecular markers, including the estrogen receptor (ER), the progesterone receptor (PgR), the epidermal growth factor receptor (EGFR), the c-erbB2 (HER2/neu) receptor, the mitotic index Ki67 and the cytokeratines 5/6 [2]. Classification of early breast cancer according to these criteria leads to five distinct immunophenotypical subtypes, namely the luminal A, luminal B, luminal-HER2, HER2-enriched and triplenegative tumor types, each one comprising a different constellation of markers [3]. Importantly, this classification was validated by large-scale genome analysis [4], [5] that demonstrated the differential gene expression signature for each immunophenotypical subtype.

The insulin and insulin-like growth factor receptor (IGFR) - mediated molecular pathways have recently emerged as important effectors of neoplastic transformation and proliferation in various malignancies [6]–[8], including breast cancer [9]. The IGFR pathway comprises two ligands (IGF1 and IGF2), their binding proteins (the most abundant being IGFBP2) and two receptors (IGF1R and IGF2R). IGF1R has the capability of signal transduction through intracellular tyrosine kinase linked to the phosphatidyl-inositol-3 kinase (PI3K)-Akt-mammalian target of rapamycin (mTOR) pathway [10]. Precursor polypeptide cleavage leads to the presence of two IGF1R isoforms: Isoform alpha (IGF1R-alpha), which is preferentially expressed in many cancers and is able to bind to insulin, IGF1 and IGF2, and isoform-beta (IGF1R-beta), which binds exclusively to insulin [10]. IGF2R, on the other hand, binds only to IGF2, is structurally distinct in the sense that it lacks an intracellular tyrosine kinase domain, and is thus deprived from the ability to transduce mitogenic signals, acting mainly as a “buffer’ for IGF2 bioactivity [11].

IGF1R has been shown to be present in all breast cancer subtypes, regardless of the hormone receptor or HER2 status, with overall IHC expression rates ranging from 43.8% [12] to 87% [9]. Critical components of the IGF1R-mediated pathway have been shown to interact with hormone receptor dependence [13], HER2 expression and resistance [14] and basal-like characteristics [15]. However, data regarding it's prognostic role in early breast cancer remain controversial, with some studies reporting an adverse impact of IGF1R overexpression on clinical outcomes [16]–[18] and others suggesting a favorable prognostic role [19]–[22]. We hypothesized that this discordance might be attributed to tumor heterogeneity and distinct molecular biology among the various breast cancer immunophenotypical subtypes. To test this hypothesis, we evaluated the protein expression of the most important components of the IGFR signaling pathway and their prognostic significance according to the tumor subtypes.

## Patient Characteristics and Methods

### Patient cohort

We collected archival tumor tissue from women with early, lymph-node positive breast cancer who were enrolled in two prospective, randomized, phase III clinical trials conducted by the Hellenic Co-operative Oncology Group (HeCOG, studies HE10/97 and HE10/00). Clinical protocols for both studies were approved by local regulatory authorities and were also included in the Australian New Zealand Clinical Trials Registry (ANZCTR) and allocated the following Registration Numbers: ACTRN-12611000506998 (HE10/97) and ACTRN-12609001036202 (HE10/00). The HE10/97 trial [23] was a randomized phase III trial in patients with high-risk node-negative or intermediate/high-risk node-positive operable breast cancer, comparing four cycles of epirubicin (E) followed by four cycles of intensified CMF (Cyclophosphamide, Methotrexate, 5-Fluorouracil, E-CMF) with three cycles of E, followed by three cycles of paclitaxel (T) followed by three cycles of intensified CMF (E-T-CMF). All cycles were given every two weeks with G-CSF support. Dose intensity of all drugs in both treatment arms was identical, but cumulative doses and duration of chemotherapy period differed. Totally, 595 eligible patients entered the study in a period of 3.5 years (1997–2000). The HE10/00 trial [24] was a randomized phase III trial, in which a total of 1,086 eligible patients with node-positive operable breast cancer were accrued in a period of 5 years (2000–2005). Patients were treated with either E-T-CMF (exactly as in the HE10/97 trial) or with four cycles of epirubicin/paclitaxel (ET) combination (given on the same day) every three weeks followed by three cycles of intensified CMF every two weeks (ET-CMF). By study design, the cumulative doses and the duration of chemotherapy period were identical in the two arms but dose intensity of epirubicin and paclitaxel was double in the E-T-CMF arm. The collection and study of tumor samples was performed in a retrospective-prospective manner (retrospectively in the HE10/97 and prospectively in the HE10/00 trial). The present translational research protocol was approved by the Bioethics Committee of the Aristotle University of Thessaloniki School of Medicine under the general title “Molecular investigation of the predictive and/or prognostic role of important signal transduction pathways in breast cancer” (A7150/18-3-2008). All patients signed a study-specific written informed consent before randomization, which in addition to giving consent for the trial allowed the use of their biological material for future research purposes. The study complied with the REMARK recommendations for tumor marker prognostic studies using biological material (available at http://www.ncbi.nlm.nih.gov/pmc/articles/PMC2361579).

For all eligible patients, clinicopathological and prospectively collected follow-up data were recorded. In both parental clinical trials, patients were followed with a physical examination, CBC, biochemistry and CA 15–3 determination, every 3 months for the first 2 years and every 6 months thereafter. Chest X-rays, ultrasonography of the abdomen and bone scans were repeated every 6 months for the first 3 years and annually thereafter. Mammography was repeated annually. Bone scans were not routinely carried out after the third year, except when clinically indicated. Other diagnostic or staging procedures were performed upon clinical indications or symptom alert.

### TMA construction

Hematoxylin-eosin stained sections from the tissue blocks were reviewed by two experienced breast cancer pathologists (M.B. and D.T.) and the most representative tumor areas were marked for the construction of the TMA blocks with the use of a manual arrayer (Model I, Beecher Instruments, San Prairie, WI). Each case was represented by 2 tissue cores, 1.5 mm in diameter, obtained from the most representative areas of primary invasive tumors or in some cases (9.6%) from synchronous axillary lymph node metastases and re-embedded in 51 microarray blocks. Each TMA block also contained cores from various neoplastic, non-neoplastic and reactive tissues, serving as controls for slide-based assays. Cases not represented, damaged or inadequate on the TMA sections were re-cut from the original blocks and these sections were used for protein analysis. Histological grade was evaluated according to the Scarff, Bloom and Richardson system.

### Immunohistochemistry

Briefly, serial 2.5 µm thick sections form the original blocks or the TMA blocks, mounted on adhesion microscope slides, were cut at the Laboratory of Molecular Oncology of the Hellenic Foundation of Cancer Research, Aristotle University of Thessaloniki School of Medicine. The immunohistochemical (IHC) labeling was performed, using Bond Max (Leica Microsystems, Wezlar, Germany) and i6000 (Biogenex, San Ramon, CA) autostainers. Samples with tumor in less than 5% of core surface were considered not interpretable and were excluded from further analysis. Immunohistochemical (IHC) staining for estrogen receptor (ER clone 6F11, Novocastra, Leica Biosystems), progesterone receptor (PgR clone 1A6, Novocastra), Ki67 (clone MIB-1, Dako, DK), cerbB2 (HER2/neu, A0485 polyclonal antibody, Dako), epidermal growth factor receptor (EGFR, clone 31G7, Invitrogen, Camarillo, CA) and cytokeratin 5 (clone XM26, Novocastra) on each slide was performed as previously described [25]. Sections were also stained with anti-IGF1R-alpha (clone 24–31, Invitrogen, at 1∶50 dilution for 1 h), anti-IGF1R-beta (C-20, sc-713, polyclonal antibody, raised against a peptide mapping at the C-terminus of the IGF-Iβ molecule, Santa Cruz, Santa Cruz, CA, at 1∶250 dilution for 1 h) and anti-IGF2R (C-15,sc-14410, goat polyclonal antibody, Santa Cruz, at 1∶250 dilution for 1 h). The antigen-antibody complex was visualized using DAB as a chromogen.

### Interpretation of the IHC results

ER and PgR positivity were defined as positive nuclear staining in at least 1% of cancer cells [26]. HER2 status was considered to be positive if HER2 was amplified (ratio >2.2 or copy number >6) by fluorescence in situ hybridization (FISH) and/or a HER2 score of 3+ was obtained by IHC [27], [28]. For Ki67, the 14% was used as cut-off to categorize low (<14%) and high (≥14%) protein status, according to Cheang et al. [29]. Any CK5 specific staining in tumor cells was considered as positive [30]. For EGFR, any membrane staining above the background in ≥1% of tumor cells was interpreted as positive [31]. Using these criteria, we assigned the patients as Luminal A (ER positive and/or PR positive and Ki-67<14%), luminal B (ER positive and/or PR positive and Ki-67≥14%), luminal/HER2 (ER positive and/or PR positive and HER2 positive), HER2 enriched (ER negative and PR negative and HER2 positive), and tripple negative (ER negative and PR negative and HER2 negative, EGFR positive or negative and CK5/6 positive or negative) [3].

**Figure 1:**
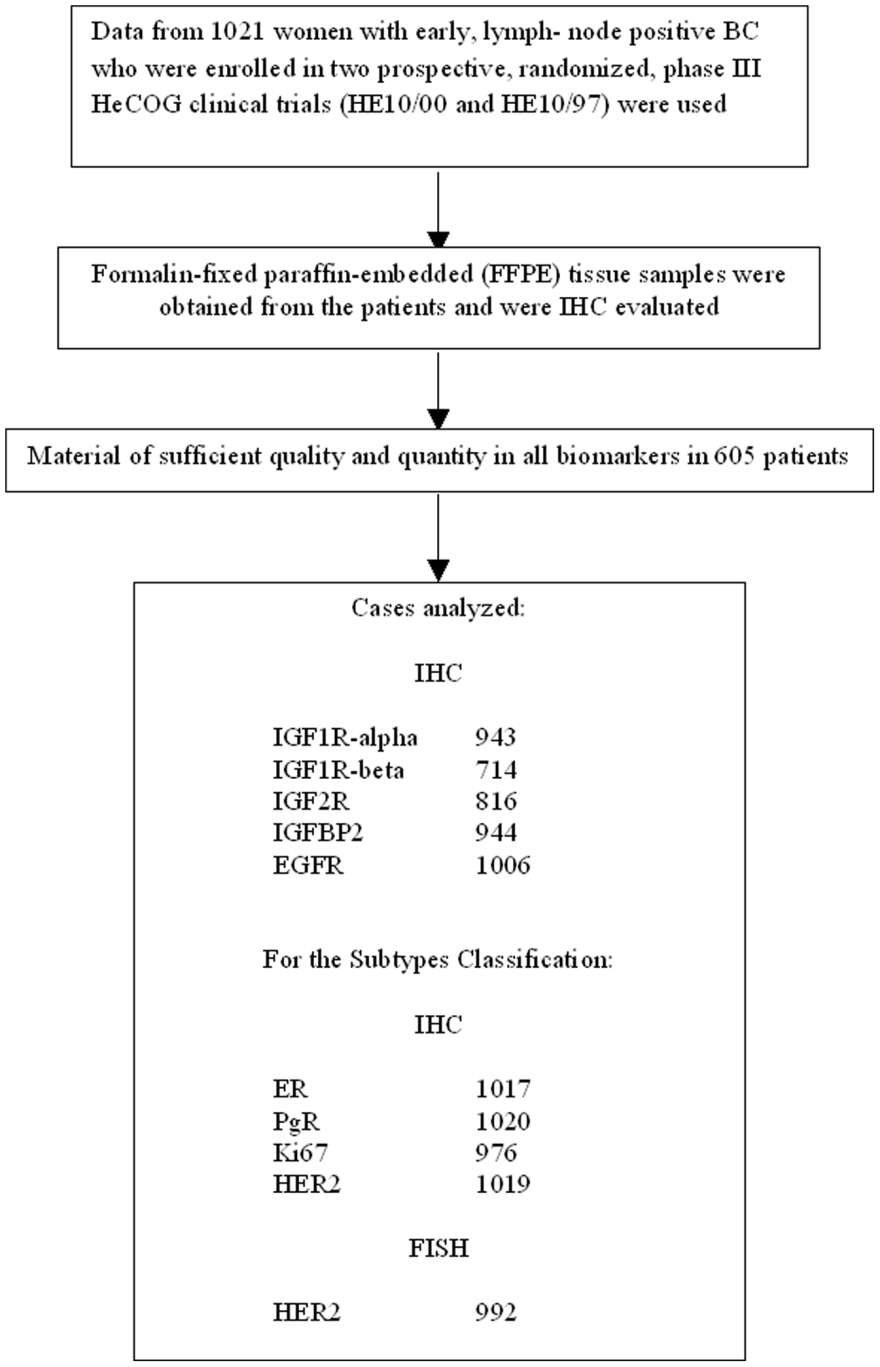
REMARK flow chart.

For the evaluation of IGF1R-alpha, IGF1R-beta and IGF2R proteins we used a semiquantitative approach based on staining intensity (SI) and percentage of positive cells (PP), to create the immunoreactive score (IRS) as follows: IRS = SIxPP, for each sample, as previously described [30]. Intensity was scored as follows: 0 = no staining, 1 = weakly positive, 2 = moderately positive and 3 = strongly positive. The scoring of the staining pattern was based on the percentage of positive tumor cells: 0 = 0%, 1 = (0–9%), 2 = (10–49%) and 3 = (50–100%). The IRS score thus ranged from 0 to 9. The localization of staining for each protein was also indicated. For the IGFBP2 evaluation the histological score (H-score) was calculated by the following method: H-score = (1×percentage of weakly positive cells)+(2×percentage of moderately strong positive cells)+(3×percentage of strongly positive cells). All discordant cases were resolved within consensus meetings. Pathologists scoring the TMA samples were blinded to the clinicopathological characteristics and outcome of each case. The flow chart of the study including the corresponding sample numbers is presented in Figure 1 (REMARK diagram).

### Statistical Considerations

Cut-off point selection was performed based on the distributional characteristics of the IRS (IGF1R-alpha, IGF1R-beta and IGF2R) and H-score (IGFBP2). For IGF1R-alpha, IGF1R-beta and IGF2R only the first, second and third quartiles were selected for further investigation since the IRS distributions where quite discrete. The distribution of H-score for IGFBP2 was granular but over-dispersed, thus, since there was no natural cut-off identified, a visual determination of a prognostic cut-off point was performed by optimizing the significance of the split of DFS and OS Kaplan-Meier plot using the 9 deciles and the logrank test. From the visual inspection of the bivariate scatter plot of each decile against the corresponding logrank p-value for IGFBP2 it was decided that the first quartile (region of the H-score distribution producing minimum p-values using the logrank test) would be a promising cut-off along with the median. The median was chosen to be explored further since the dichotomized biomarker in the first quartile might result either in great loss of power or in aberrant results in the analysis by subtype due to the reduction in the available sample size for analysis (i.e. below the cut-off point). The figures of the distributions of the IRS (IGF1R-alpha, IGF1R-beta and IGF2R) and H-score (IGFBP2) along with the bivariate scatter plots of the logrank p-value against the 9 deciles for all the biomarkers can be found in Figure S1, A–D and E–H respectively.

In order to avoid false-positive findings arising from multiple cut-off calculations, we used an internal validation method in order to assess the statistical validity of the candidate cut off points for all biomarkers. The total sample of 1,021 patients was split in two datasets (training and validation) 100 times controlling for the following parameters to be equally assigned in the two sets: nodal status, type of surgery, immunophenotypical subtype and treatment with taxanes. Using the training set to compute the median values for the scores for each replication, and considering that “high-expression” of the IRS or H-score was above or equal to the median and “low-expression” otherwise, we assigned the cut off points to the validation set. For each replication hazard ratios were computed, using Cox-regression models, and the mean differences between training and validation sets were calculated, as well as the percent where the upper or lower limit of the 95% confidence intervals that crossed 1. The median value of the medians was used as the pre-defined cut-off for each marker for the whole dataset, as previously suggested [32]. The cross validation analysis was repeated for the first and third quartiles but neither candidate could be considered further as a valid cut-off point.

Multiple correspondence analysis, as a descriptive/exploratory technique, was used in order to reveal latent patterns of dependencies between the biomarkers, patterns which are masked when bivariate correlations are examined.

DFS was measured from the time of diagnosis until verified disease progression, death or last contact and OS from diagnosis until death from any cause or date of last contact. Time-to-event distributions were estimated using Kaplan-Meier curves. Associations between biomarkers and with basic patient and tumor characteristics were examined using the Fisher's exact test for categorical variables and the Mann-Whitney or the Kruskall-Wallis tests where appropriate for continuous variables. Log-rank tests and univariate Cox analysis were used for evaluating DFS and OS differences and reporting hazard ratios, respectively.

In multivariate analysis, using Cox regression, significance was determined at the level of 15% and in univariate at 5% (two-sided). All tests were two-sided. Two separate multivariate analysis models are reported in order to offset multicollinearity effects caused by the high correlation of the IGF1R-alpha/IGF2R variable with the IGF1R-alpha/EGFR variable.

The SAS software was used for statistical analysis (SAS for Windows, version 9.2, SAS Institute Inc., Cary, NC, USA), while no adjustment for multiple comparisons is reported. The statistical analysis complied with the reporting recommendations for tumor marker prognostic studies [33].

## Results

### Clinicopathological characteristics and outcome

Valid archival tumor samples were available from one thousand and twenty-one patients (N = 1,021) from both clinical trials and were included in the present analysis. As shown in Table 1, basic clinicopathological characteristics were representative of the typical patient population with early, node-positive breast cancer, with 53.6% of women being post-menopausal, 31.4% having undergone breast-conserving surgery, 77.8% bearing hormone-receptor positive tumors and 75.6% having received post-operative radiotherapy. Regarding subgroup classification, 252 cases (24.6%) were classified as luminal A type, 394 (38.6%) as luminal B, 137 (13.4%) as luminal-HER2, 109 (10.6%) as HER2-enriched and 129 cases (12.6%) as “triple negative”.

**Table 1:** Clinicopathological characteristics of the whole patient cohort and by immunophenotypical subtypes.

			Subtypes Classification					
		Total Sample	Luminal A	Luminal B	Luminal-HER2	HER2-enriched	Triple negative	P- value
**Patients**	**N**	**1,021**	**252**	**394**	**137**	**109**	**129**	
Age (years)	Median	52.7	55.1	52.1	49.3	54.3	52.7	0.003
	Min-Max	22–79	22–79	25–78	24–79	25–77	22–75	
Adjuvant	No	205 (20%)	23 (9.2%)	42 (10.6%)	18 (13.2%)	57 (52.2%)	65 (50.4%)	<0.001
Hormonotherapy	Yes	794 (77.8%)	222 (88%)	342 (86.8%)	116 (84.6%)	52 (47.8%)	62 (48%)	
	Not reported	22 (2.2%)	7 (2.8%)	10 (2.6%)	3 (2.2%)		2 (1.6%)	
Adjuvant Radiotherapy	No	218 (21.4%)	63 (25%)	87 (22%)	22 (16%)	21 (19.2%)	25 (19.4%)	0.285
	Yes	771 (75.6%)	179 (71%)	298 (75.6%)	109 (79.6%)	85 (78%)	100 (77.6%)	
	Not reported	32 (3.2%)	10 (4%)	9 (2.2%)	6 (4.4%)	3 (2.8%)	4 (3.2%)	
Histological grade	I–II	507 (49.6%)	188 (74.6%)	195 (49.4%)	59 (43%)	26 (23.8%)	39 (30.2%)	<0.001
	III-Undifferentiated	514 (50.4%)	64 (25.4%)	199 (50.6%)	78 (57%)	83 (76.2%)	90 (69.8%)	
Histology classification recoded	Mixed	74 (7.2%)	20 (8%)	39 (9.8%)	7 (5.2%)	6 (5.6%)	2 (1.6%)	<0.001
	Invasive ductal	792 (77.6%)	182 (72.2%)	293 (74.4%)	117 (85.4%)	97 (89%)	103 (79.8%)	
	Invasive lobular	105 (10.2%)	40 (15.8%)	45 (11.4%)	8 (5.8%)	2 (1.8%)	10 (7.8%)	
	Other	50 (4.8%)	10 (4%)	17 (4.4%)	5 (3.6%)	4 (3.6%)	14 (10.8%)	
Interval from operation	<2 weeks	96 (9.4%)	25 (10%)	39 (9.8%)	10 (7.2%)	9 (8.2%)	13 (10%)	0.568
	2–4 weeks	461 (45.2%)	101 (40%)	182 (46.2%)	72 (52.6%)	50 (45.8%)	56 (43.4%)	
	>4weeks	462 (45.2%)	126 (50%)	171 (43.4%)	55 (40.2%)	50 (45.8%)	60 (46.6%)	
	Not reported	2 (0.2%)		2 (0.6%)				
Menopausal Status	Pre	474 (46.4%)	108 (42.8%)	191 (48.4%)	70 (51%)	47 (43.2%)	58 (45%)	0.441
	Post	547 (53.6%)	144 (57.2%)	203 (51.6%)	67 (49%)	62 (56.8%)	71 (55%)	
Positive lymph nodes	0	4 (0.4%)		1 (0.2%)	2 (1.4%)		1 (0.8%)	0.026
	1–3	399 (39%)	121 (48%)	143 (36.2%)	49 (35.8%)	37 (34%)	49 (38%)	
	>4	618 (60.6%)	131 (52%)	250 (63.4%)	86 (62.8%)	72 (66%)	79 (61.2%)	
Tumor size	≤2 cm	315 (30.8%)	88 (35%)	121 (30.8%)	39 (28.4%)	26 (23.8%)	41 (31.8%)	0.300
	>2 cm	706 (69.2%)	164 (65%)	273 (69.2%)	98 (71.6%)	83 (76.2%)	88 (68.2%)	
Surgery	MRM	700 (68.6%)	175 (69.4%)	277 (70.4%)	90 (65.6%)	83 (76.2%)	75 (58.2%)	0.032
	Breast conserving surgery	321 (31.4%)	77 (30.6%)	117 (29.6%)	47 (34.4%)	26 (23.8%)	54 (41.8%)	

Note: The p-values correspond to Kruskal-Wallis test for age and Chi-square test for the other categorical variables. MRM: Modified radical mastectomy.

After a median follow-up time of 105.4 months (range: 0.1–166.7), overall 370 women (36.2%) had relapsed and 270 (26.4%) had died. The median and 4-year DFS were 160 months (95% CI: 158–Not reached) and 76.1%, respectively. The median OS had not been reached and 4-year OS rate was 89.5%. As expected, 4-year OS rates were significantly lower for patients with hormone receptor negative disease as compared to those with hormone receptor positive (83.3% vs. 91.1%, p = 0.0099), for patients with high-grade tumors (grade III) as compared to those with grade I and II (86.6% vs. 92.4%, p = 0.0085) and for patients who received post-operative radiotherapy as compared to those who did not (88.6% vs. 92.5%, p = 0.0099), probably due to the fact that the latter group had limited lymph-node involvement and smaller tumor size compared to the former. Finally, there was no effect of the treatment regimen (taxane-containing versus no taxane-containing) on clinical outcomes.

### Immunohistochemistry and distribution according to tumor subtypes

**Figure 2:**
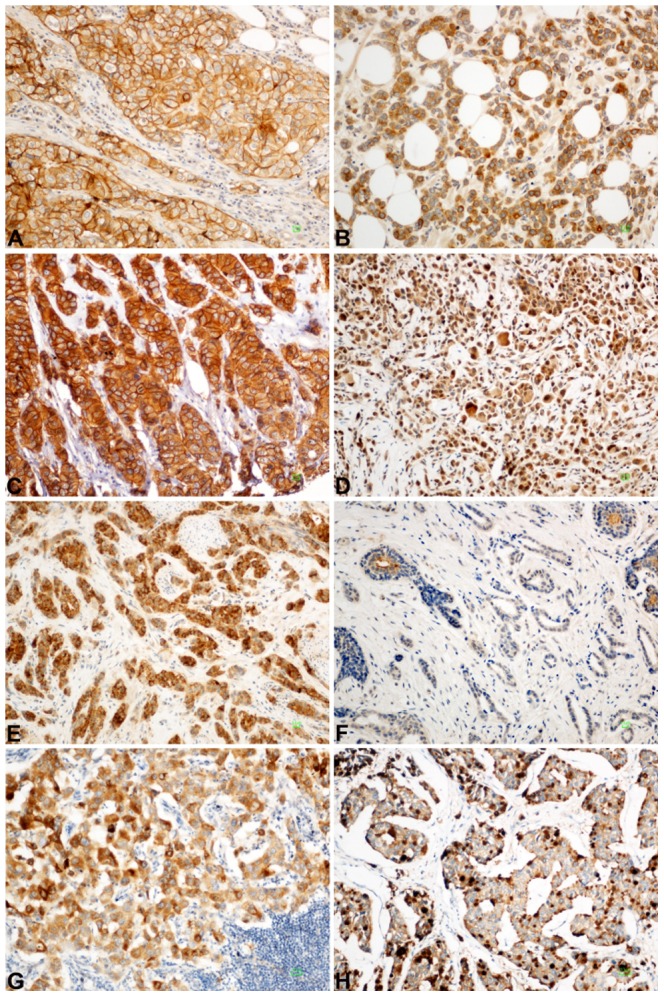
Protein expression detected by IHC from invasive breast carcinoma cases. (**A**) IGF1R-alpha moderate to strong membraneous staining in carcinoma cells; (**B**) IGF1R-alpha cytoplasmic staining; (**C**) IGF1R-beta intense cytoplasmic and membraneous staining; (**D**) IGF1R-beta cytoplasmic expression in neoplastic population; (**E**) IGF2R, predominantly granular cytoplasmic staining; (**F**) IGF2R expression limited to non-neoplastic epithelial remnants; (**G**) IGFBP2 moderate to intense cytoplasmic staining in neoplastic cells; (**H**) IGFBP2 cytoplasmic and dot-like pattern of staining. Scale Bar: 10 µm.

**Figure 3:**
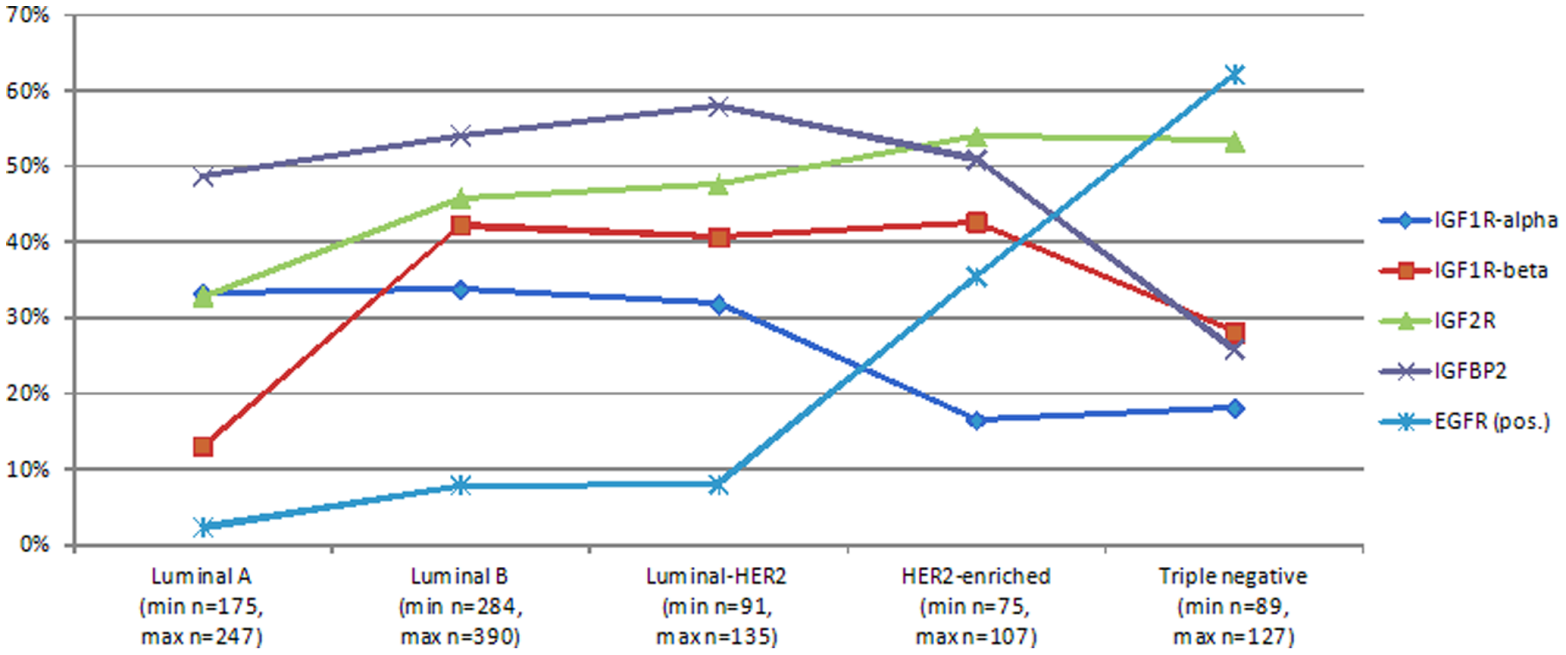
Line graph for high (or positive) levels of the biomarkers.

The IHC staining for IGF1R-alpha was mainly cytoplasmic and/or membranous and was moderate or strong in 54.4% of cases (median IRS = 2, Figures 2A+2B). As illustrated in Figure 3, tumors overexpressing IGF1R-alpha (i.e. above the median IRS) were more frequently hormone-sensitive, including the luminal A, luminal B and luminal-HER2 subtypes, as compared to HER2-enriched and triple negative tumors that overexpressed IGF1R-alpha in only 16.5% and 18.1% of cases, respectively (p<0.001 for both comparisons). On the contrary, IGF1R-beta staining was pre-dominantly cytoplasmic and was absent (IRS = 0) in 66.8% of cases (Figures 2C and 2D). These findings urged us to further study expression of IGF1R-alpha in patients with hormone-receptor positive disease in particular.

The staining pattern for type 2 IGF receptor (IGF2R) was present in 55.5% of cases and was predominantly cytoplasmic (Figures 2E and 2F) but, in contrast with IGF1R, it was overexpressed significantly more frequently in hormone receptor negative tumors (including the HER2-enriched and the triple negative tumors) as compared to the hormone sensitive ones (p = 0.001, Figure 3), suggesting a preferential expression of IGF2R in hormone receptor negative tumors. IGFBP2 staining (Figures 2G and 2H), on the other hand, exhibited no differential expression across the different tumor subtypes, with the exception of significantly lower expression in triple negative tumors as compared to all the other groups (p<0.001).

EGFR was negative in the majority of tumors (82.4%), but, as expected, it was significantly higher in the triple negative and the HER2-enriched groups, as compared to the group of hormone receptor positive tumors (p<0.001 for both comparisons). Triple negative tumors, in particular, expressed EGFR significantly more than did HER2-enriched ones (62.2% vs. 35.5%, p<0.001, Figure 3).

### Correlations between biomarkers and with clinical outcomes

**Figure 4:**
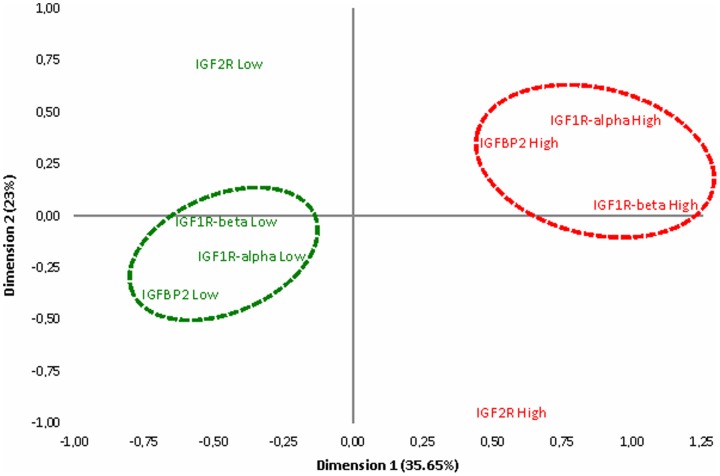
Multiple correspondence analysis graph.

“Using multiple correspondence analysis, we identified strong correlations between IGF1R-alpha, IGF1R-beta, IGF2R and IGFBP2 IHC expression accounting for the 35.65% of total inertia explained by the analysis (illustrated in Figure 4 and Table S3)”. However, IGF2R seemed to have an inverse correlation in the remaining 23% of total inertia with all other components of the IGFR pathway, reinforcing its presumed role as a “suppressor” of the IGF1R axis.

In univariate analysis, none of the components of the IGFR-mediated pathway was able to predict clinical outcome (DFS or OS) in the whole patient population (data not shown). However, the fact that IGF1R-alpha was inversely correlated with IGF2R (Figure 4) and that it was overexpressed mostly in hormone receptor positive tumors, whereas EGFR was expressed mostly in hormone receptor negative tumors, urged us to study the following two clusters of tumors that indicate an aberrant expression of the IGFR pathway: a) Hormone receptor positive tumors with high IGF1R-alpha and low IGF2R expression and b) Hormone receptor positive tumors with high IGF1R-alpha and negative EGFR expression.

**Figure 5:**
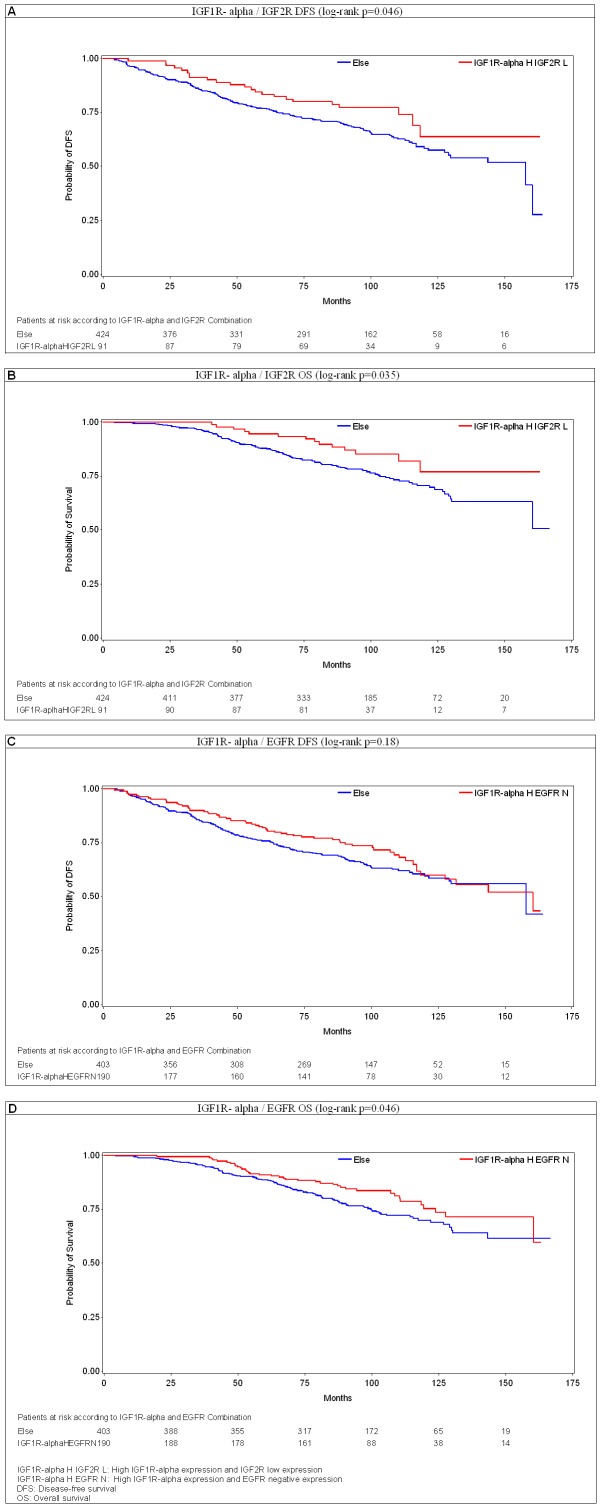
Kaplan - Meier Curves for the IGF1R- alpha and IGF2R combination variable- DFS (patients of luminal A and luminal B subtype) in panel A. B: Kaplan- Meier Curves for the IGF1R- alpha and IGF2R combination variable- OS (patients of luminal A and luminal B subtype).

In the first cluster, patients with luminal A and B tumors with high IGF1R-alpha and low IGF2R expression (N = 91) experienced significantly longer DFS as compared to the rest of the patient population (4-year DFS rates: 87.8% vs. 80.2% respectively, log-rank p = 0.046, univariate Cox HR = 0.642, 95% CI: 0.414–0.995, p = 0.048) and significantly longer OS (4-year OS rates: 97.8% vs. 91.4% respectively, log-rank p = 0.035, univariate Cox HR = 0.555, 95% CI: 0.318–0.968, p = 0.038) (Figures 5A and 5B). When adding luminal-HER2 patients in the cluster, the effect of the IGF1R-alpha and IGF2R combination on DFS was not significantly altered (HR = 0.718, 95% CI: 0.488–1.055, p = 0.092, interaction test p = 0.191) while on OS it was (HR = 0.725, 95% CI: 0.457–1.148, p = 0.171, interaction test p = 0.017). This result indicates a significant effect of HER2 status on OS in hormone receptor positive tumors with aberrant expression of the IGFR pathway.

In the second cluster, patients with luminal A and B tumors with high IGF1R-alpha and negative EGFR expression (N = 190), had a trend, albeit not significant, for longer DFS as compared to the rest of the patient population (4-year DFS rates: 85.2% vs. 79.4% respectively, log-rank p = 0.186, univariate Cox HR = 0.818, 95% CI: 0.607–1.10, p = 0.186) but had significantly longer OS (4-year OS rates: 96.3% vs. 90.9% respectively, log-rank p = 0.046, univariate Cox HR = 0.688, 95% CI: 0.475–0.997, p = 0.048), (Figures 5C and 5D). Again, when adding the luminal-HER2 patient group, the effect of the IGF1R-alpha and EGFR combination on DFS was not altered (HR = 0.845, 95% CI: 0.648–1.101, p = 0.212, interaction test p = 0.593) while on OS it was (HR = 0.802, 95% CI: 0.583–1.102, p = 0.174, interaction test p = 0.057– marginally statistically significant). Moreover, the study of all other possible combinations of these markers (IGF1R low/IGF2R low, IGF1R high/IGF2R high, IGF1R high/EGFR high and IGF1R low/EGFR low) did not reveal any prognostic significance on DFS and/or OS for any cluster (data not shown). This was expected since: a) IGF2R is considered to be a suppressor of the IGFR pathway and thus the clusters IGF1R low/IGF2R low and IGF1R high/IGF2R high do not indicate aberrant expression of the IGFR pathway and b) EGFR was overexpressed mainly in hormone receptor negative patients and thus the clusters IGF1R high/EGFR high and IGF1R low/EGFR low are not expected to correlate with aberrant IGFR expression in Luminal A and B tumors, which are hormone receptor positive. Taken altogether, these results suggest that aberrant expression of components of the IGF1R-mediated pathway is associated with better clinical outcomes in women with hormone receptor positive, HER2 negative, node positive early breast cancer.

### Internal cross-validation results

For the three variables quantified with the IRS (IGF1R-alpha, IGF1R-beta, IGR2R) and the single variable quantified with the H- score (IGFBP2), we computed the median values in the training set (N = 522) for each one of the 100 replications *(see statistical considerations session)* and then we applied them in the validation set (N = 499) by splitting the variables into high and low levels (Table S1).

Using these binary variables and the EGFR binary - positive vs. negative (<1%) in all replications and in both datasets we computed the corresponding Hazard Ratios (HRs) for each replication, dataset and clinical endpoint (DFS/OS). The whole procedure revealed that none of the markers was independently prognostic in terms of DFS or OS in the whole study population, since almost all (in 85%–100% of the replications) of the 95%CIs of the HRs computed crossed the value 1, while the estimates oscillated around 1 (Figure S2, A–D). Nevertheless, combined EGFR with IGF1R-alpha is a candidate prognostic marker for OS, while IGF1R-alpha with IGF2R is a candidate prognostic marker for both OS and DFS in Luminal A and B patients, since even though almost all (in 80%–86% of the replications) of the 95%CIs of the computed HRs still crossed 1, the majority (the upper limit of the interquartile range of the HR point estimates was 0.933) of the estimates were under 1 (Figure S2, E–H). This result occurred in the whole patient dataset and particularly in the only Luminal A and B dataset (Table S2).

### Cox regression analysis

After adjustment for all significant clinicopathological variables, the IGF1R-alpha/IGF2R combined variable was independently associated with clinical outcomes; As shown in Table 2, patients bearing tumors with high IGF1R-alpha and low IGF2R expression, had a relative 45% reduction in the risk of death, as compared to the rest of the patient population (HR = 0.55, 95% CI: 0.31–0.96, p = 0.035) and the IGF1R-alpha/IGF2R variable was the third most powerful prognosticator for survival, following the number of positive lymph nodes (>4 vs. 0–3) and the type of surgery (modified radical mastectomy vs. breast-conserving surgery). The same variable was also marginally associated with better DFS (HR = 0.65, 95% CI: 0.42–1.01, p = 0.056). Regarding the IGF1R-alpha/EGFR variable, the combination of high IGF1R-alpha expression and negative EGFR expression was marginally associated with superior OS (HR = 0.69, 95% CI: 0.48–1.01, p = 0.054) and not significantly associated with prolonged DFS (HR = 0.78, 95% CI: 0.58–1.05, p = 0.104, Table 3).

**Table 2:** Multivariate model for the IGF1R-alpha and IGF2R combined variable adjusted for clinical parameters (patients of luminal A and B subtype only).

IGF1R-alpha and IGF2R	Disease Free Survival (N = 515)			Overall Survival (N = 515)		
	HR	95% CI	Wald's p	HR	95% CI	Wald's p
**Surgery**						
Breast conserving surgery vs. MRM	0.59	0.41–0.85	0.0043	0.50	0.32–0.79	0.0032
**Nodes positive**						
0–3 vs. > = 4	0.54	0.39–0.76	0.0003	0.46	0.30–0.70	0.0002
**IGF1R-alpha and IGF2R Combined variable**						
IGF1Ra High and IGF2R Low vs. Else	0.65	0.42–1.01	0.0560	0.55	0.31–0.96	0.0345

**Table 3:** Multivariate model for the IGF1R-alpha and EGFR combined variable adjusted for clinical parameters (patients of luminal A and B subtype only).

IGF1R-alpha and EGFR	Disease Free Survival (N = 575)			Overall Survival (N = 593)		
	HR	95% CI	Wald's p	HR	95% CI	Wald's p
**Size**						
< = 2 cm vs. >2 cm	-	-	-	0.73	0.49–1.07	0.1033
**Surgery**						
Breast conserving surgery vs. MRM	0.54	0.38–0.77	0.0006	0.57	0.38–0.87	0.0089
**Nodes positive**						
0–3 vs. > = 4	0.65	0.46–0.92	0.0166	0.45	0.30–0.66	<.0001
**Histology classification**						
Invasive ductal vs. Mixed	0.64	0.43–0.96	0.0293	-	-	-
Invasive lobular vs. Mixed	0.59	0.34–1.00	0.0484	-	-	-
Other vs. Mixed	0.28	0.10–0.80	0.0176	-	-	-
**Adjuvant Radiotherapy**						
No vs. Yes	0.71	0.47–1.08	0.1101	-	-	-
**IGF1R-alpha and EGFR Combined variable**						
IGF1Ra High and EGFR Negative vs. Else	0.78	0.58–1.05	0.1045	0.69	0.48–1.01	0.0540

HR: hazard ratio.

MRM: Modified radical mastectomy.

## Discussion

In the current study, one of the largest in our knowledge to assess IGFR protein expression in early breast cancer, we found an overall moderate or strong expression of IGF1R-alpha in 54.4% of the whole patient population, which is consistent with previous studies reporting expression rates of 43.8% [12] to 87% [19]. IGF1R-alpha is the main receptor subunit that is able to activate both pERK- and pAkt-mediated downstream signaling pathways [13] and has been implicated in breast carcinogenesis [34], miRNA regulation in HR positive breast cancer cell lines [35] endocrine resistance [36] and aromatase inhibitor-related symptoms [37], suggesting a cross-talk of the IGF receptor with hormone receptors. Our results confirm this strong association, since IGF1R-alpha was consistently overexpressed in all hormone-receptor positive tumors (including the luminal-HER2 subtype), as compared to hormone-receptor negative ones. Regarding its prognostic role in early breast cancer, IGF1R-alpha has been associated with adverse clinical outcomes in some studies [16]–[18] and with favorable prognosis in others [19]–[22]. We found that aberrant expression of components of the IGFR pathway was associated with better clinical outcomes only in patients with hormone-receptor positive, HER2-negative tumors. These results suggest that the prognostic role of the IGFR pathway differs according to the immunophenotypical subtype of breast cancer and may explain, at least in part, the conflicting results of previous studies that comprised different subtype populations. For example, in the study by *Nielsen et al.* [18], which reported a correlation between high IGF1R expression and poor survival in a cohort of 930 patients, only half of the tumors were hormone-receptor positive, whereas in our cohort this percentage was substantially higher (75%). Moreover, the same study [18] included also patients with lymph-node negative disease (29%), while our cohort included almost exclusively patients with node-positive disease, and this may explain the significantly higher expression of IGF1R in the study by *Nielsen et al.* as compared to our results (87.0% vs. 54.4% respectively), since higher IGF1R expression has been correlated with node-negative disease [22]. The favorable prognostic role of IGFR is further supported by studies on IGF1R mRNA expression, which has been correlated with better clinical outcomes, especially in the luminal subtype groups [22].

In a recent important study in the field, *Yerushalmi et al.* [9] reported that IGF1R overexpression was associated with better breast cancer-specific survival (BCSS) in patients with luminal B tumors, which is in accordance with our results, and with worse BCSS in patients with HER2-enriched tumors, which was not confirmed in our cohort. Again, possible reasons for this discordance should be sought with regard to patient population heterogeneity: In the study by *Yerushalmi et al.* [9], almost half of the patients had node-negative disease, as compared to only 0.4% in our cohort; Moreover, the monoclonal antibody used in the same study [9] was not directed specifically against the alpha subunit of IGF1R and the methodology used for quantification of IGF1R immunohistochemical expression (Alred score) was different from the one used in the current study (IRS). Importantly, increased IGF1R-mediated signaling has been related to resistance to anti-HER2 therapies including trastuzumab [38] and has been proposed as a possible therapeutic target [14] and a prognostic marker [39] in HER2-positive tumors. More recently, accumulating data implicate IGFR pathway in the pathogenesis of triple-negative tumors [15], [40], as well as in tumors with BRCA1 mutations [41], [42].

We found that IGF2R was inversely correlated with all other components of the IGFR pathway (Figure 4) and that low IGF2R expression, combined with high IGF1R-alpha expression, were able to define a distinct subgroup of patients with better prognosis. These results further support the presumed role of IGF2R as a “buffer” for IGF2 bioactivity, limiting the binding of IGF2 to IGF1R and reducing thus activation of the downstream pathway [10]. It is therefore reasonable to postulate that the combination of high IGF1R and low IGF2R expression is suggestive of possible aberrant expression of the IGFR-mediated pathway. In the same context, EGFR was found to be more frequently expressed in triple negative and HER2-enriched tumors (62.2% and 35.5% respectively), which represent the two molecular subtypes with the lower frequency of IGF1R-alpha expression (18.1% and 16.5%, respectively). Inversely, IGF1R-alpha expression was much more evident in hormone-receptor positive tumors (more than 30% in each of the three categories: luminal A, luminal B and luminal-HER2), whereas EGFR expression was practically absent in the same molecular categories (less than 10% in each), suggesting a possible compensatory mechanism of IGF1R-mediated signaling in tumors lacking EGFR-mediated signaling (illustrated in Figure 3). This hypothesis is supported by preclinical data reporting important cross talk between the two pathways, especially in hormone-receptor positive tumors [13].

The insulin-like growth factor binding proteins (IGFBP) are important regulators of the IGFR pathway, acting mainly by binding to the ligands IGF1, IGF2 and insulin, but they are also directly implicated in the process of carcinogenesis: IGFBP3 is involved in the regulation of DNA damage response [43] and IGFBPs 4 and 5 may prevent hormone-dependent activation of estrogen-receptor positive breast cancer cell growth in an IGF1R-independent manner [44]. IGFBP2, which was measured in the current study, has been reported to act as a potent mitogenic, by enhancing the proliferative capacity of breast cancer cells, protecting them from chemotherapy-induced apoptosis, and maintaining estrogen-receptor expression [45]. However, their clinical validity may be limited, especially when measured as plasma concentrations, mainly due to reasons of biological variability [46].

Our study has some limitations: The collection and study of tumor samples was performed in a retrospective-prospective manner (retrospectively in the HE10/97 and prospectively in the HE10/00 trial), however, the pathological review of each case and the subsequent molecular allocation were done by central review and data regarding clinical outcomes were derived from prospective clinical trials with strict protocol criteria regarding evaluation of clinical endpoints. Second, evaluation of immunohistochemical tissue microarray blocks using semi-quantitative procedures such as the IRA and the H-score, is prone to subjectivity and may not accurately reflect expression in the whole tumor sample, which may have hampered correlation evaluations. Reproduction and validation of these results will require robustly-designed and well-conducted prospective trials incorporating evaluation of the appropriate biomarkers in biological samples obtained during and after the enrolment of patients.

In conclusion, we found that aberrant expression of important components of the IGFR-mediated signaling pathway, and especially the IGF1R-alpha/IGF2R combination, are associated with better clinical outcomes in patients with hormone-receptor positive, HER2-negative, node-positive early breast cancer. These results further support the important interplay between the IGFR pathway and hormone receptors and suggest a potential role for the elements of this pathway as molecular targets for therapeutic intervention in hormone-receptor positive disease. Early clinical trials employing monoclonal antibodies against IGF1R are currently underway in a variety of solid tumors including breast cancer [47], [48]. If our results are validated by large prospective clinical trials, evaluation of the IGFR pathway will offer important prognostic and therapeutic opportunities in patients with early breast cancer in the near future.

## Supporting Information

Figure S1 **Computed Hazard Ratios (HR) for the 100 splits of the training and validation sets evaluating the two biomarker clusters (IGF1R/IGF2R and IGF1R/EGFR in the whole study population (1A–1D) and the Luminal A and B patient cohort (1E–1H).**
(PPT)

Figure S2 **Computed corresponding Hazard Ratios (HRs) for each replication and clinical endpoint (DFS/OS), for the whole study population (A–D) and the Luminal A and B patient cohort (E–H).**
(PPT)

Table S1 **Identification of the cut-off values for each biomarker in the training set using 100 random splits.**
(XLS)

Table S2 **Mean and median differences in Hazard Ratios (HR) of the IGF1R/IGF2R and IGF1R/EGFR clusters in the 100 splits of the training and validation set in the whole study population (A) and in Luminal A and B patients (B).**
(PPT)

Table S3 **Bivariate associations between immunohistochemical expression of biomarkers.**
(PPT)
