# Supplementary material for: Differential Expression of the Insulin-Like Growth Factor Receptor among Early Breast Cancer Subtypes
Source: PLoS One. 2014 Mar 17;9(3):e91407. doi: 10.1371/journal.pone.0091407 (PMC3956672; doi:10.1371/journal.pone.0091407)
Supplement: Figure S1 — Computed Hazard Ratios (HR) for the 100 splits of the training and validation sets evaluating the two biomarker clusters (IGF1R/IGF2R and IGF1R/EGFR in the whole study population (1A–1D) and the Luminal A and B patient cohort (1E–1H). (PPT) [file pone.0091407.s001.ppt]

## Slide 1
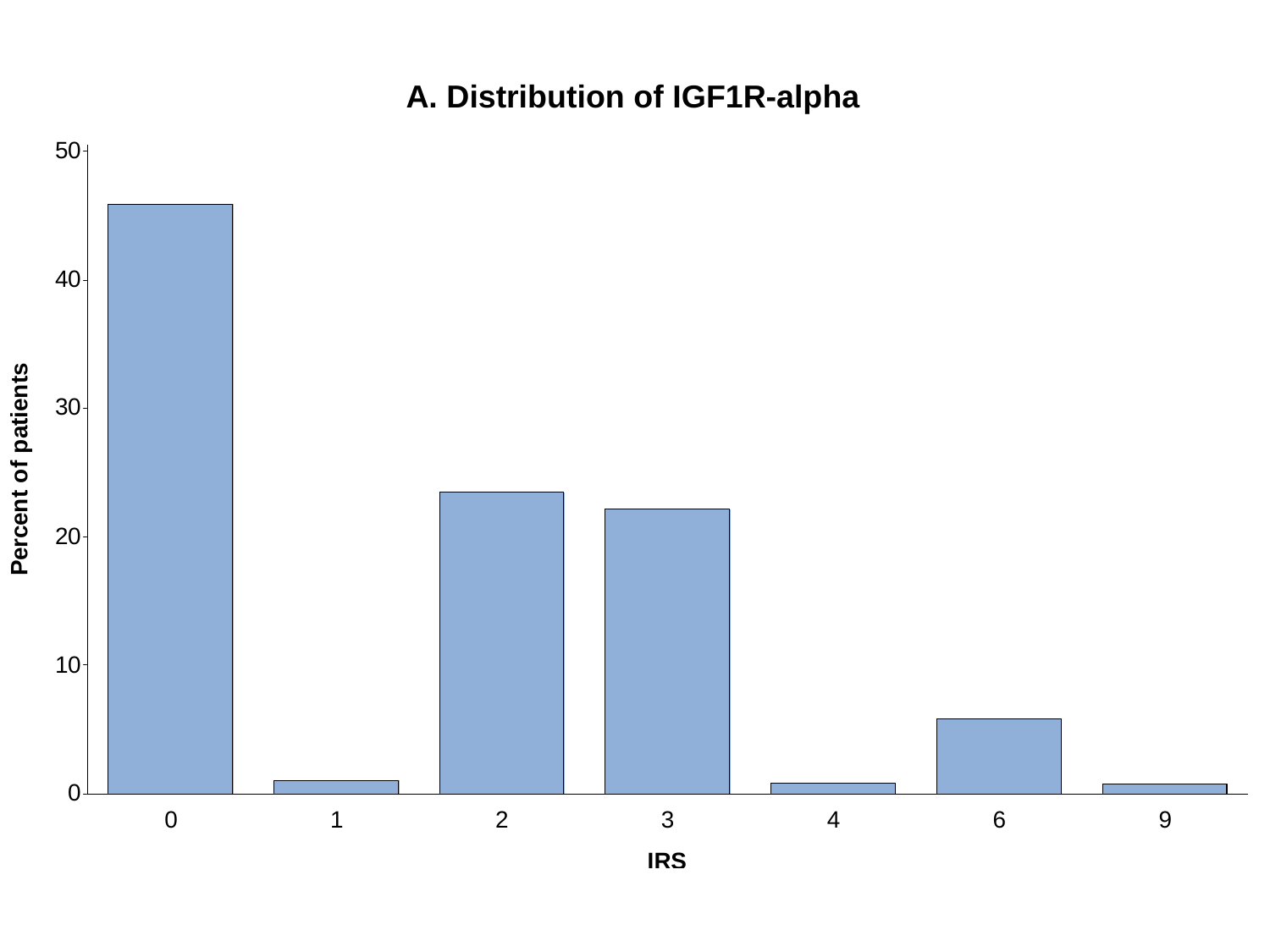

A. Distribution of IGF1R-alpha

## Slide 2
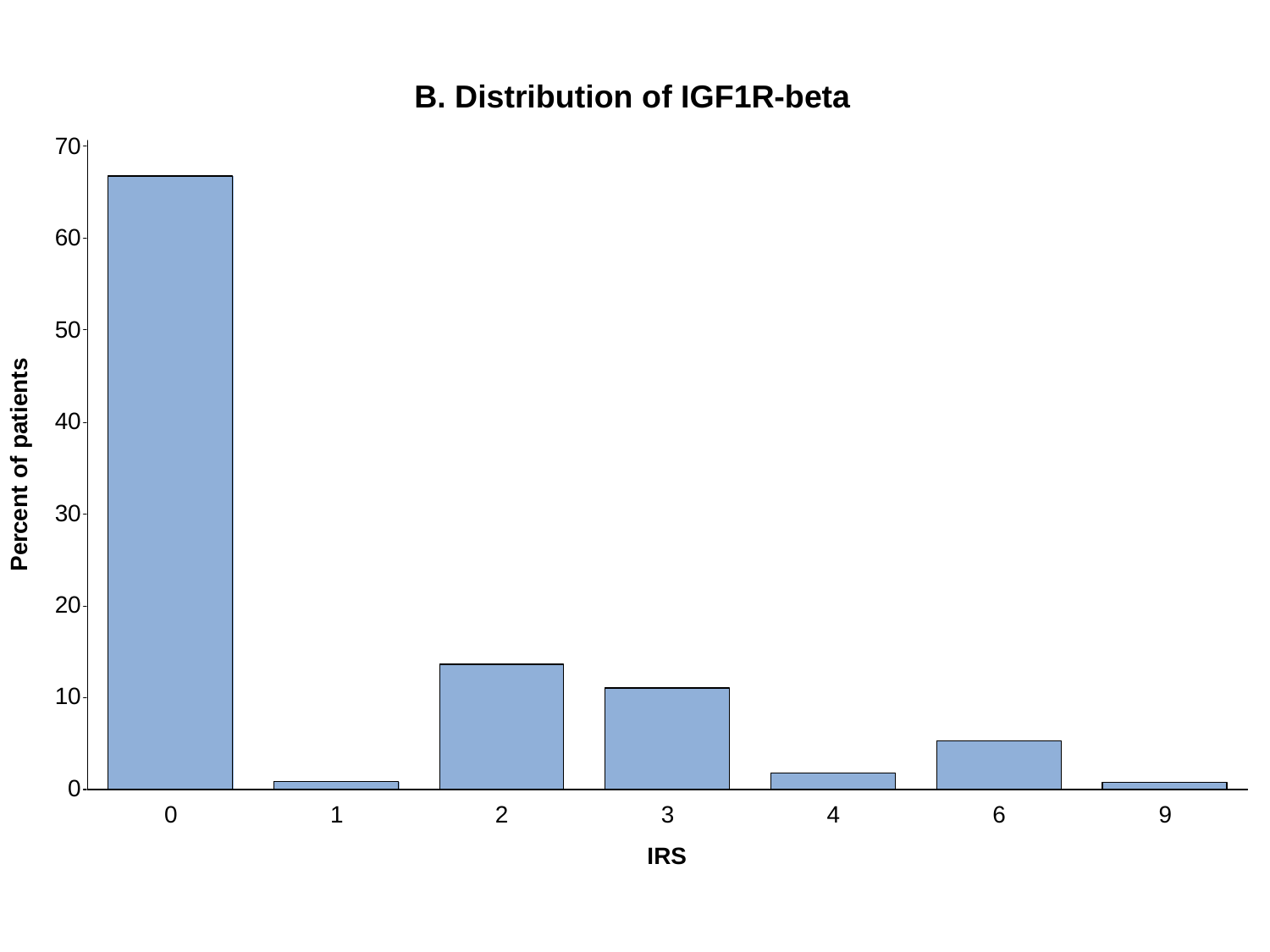

B. Distribution of IGF1R-beta

## Slide 3
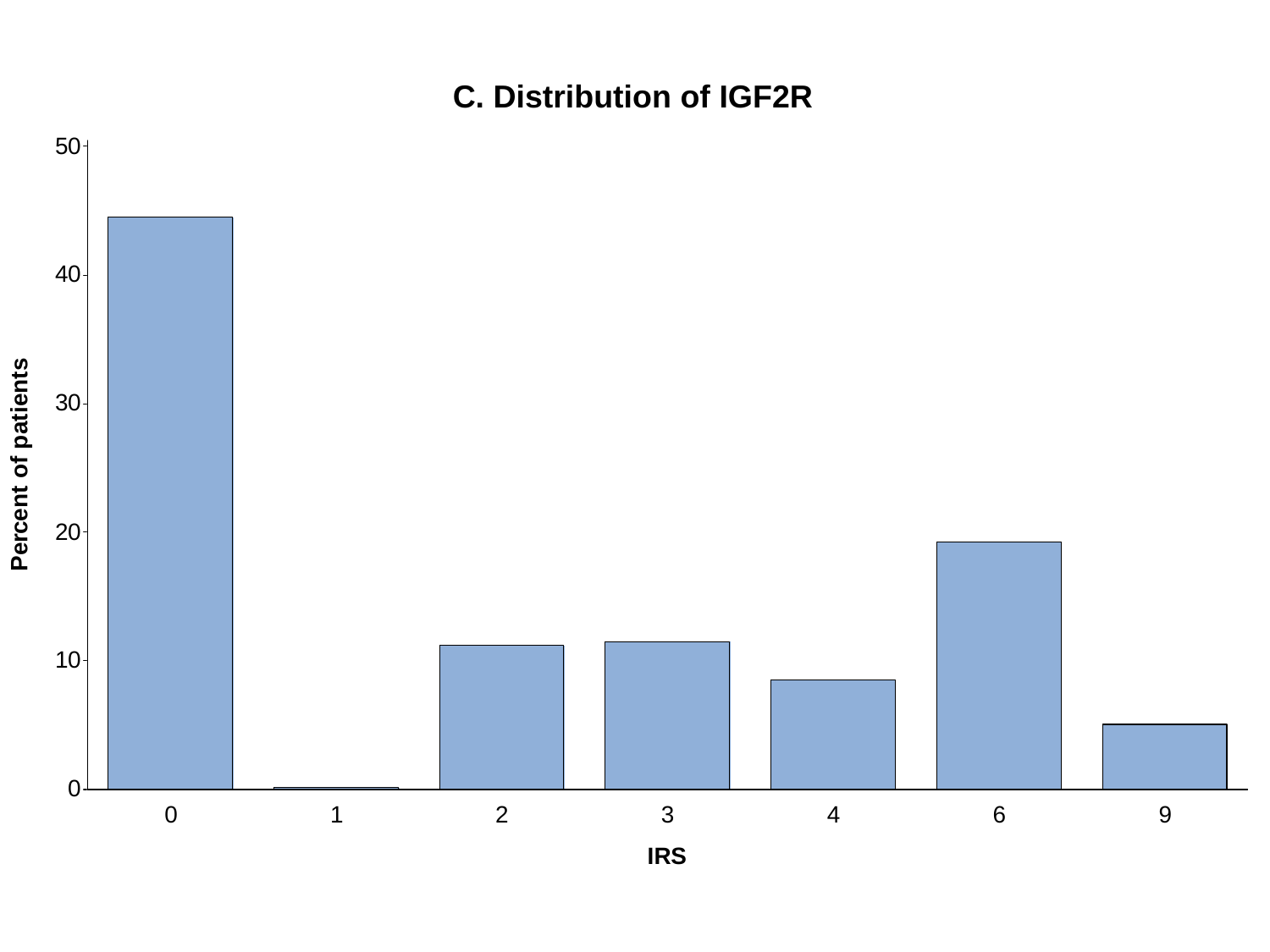

C. Distribution of IGF2R

## Slide 4
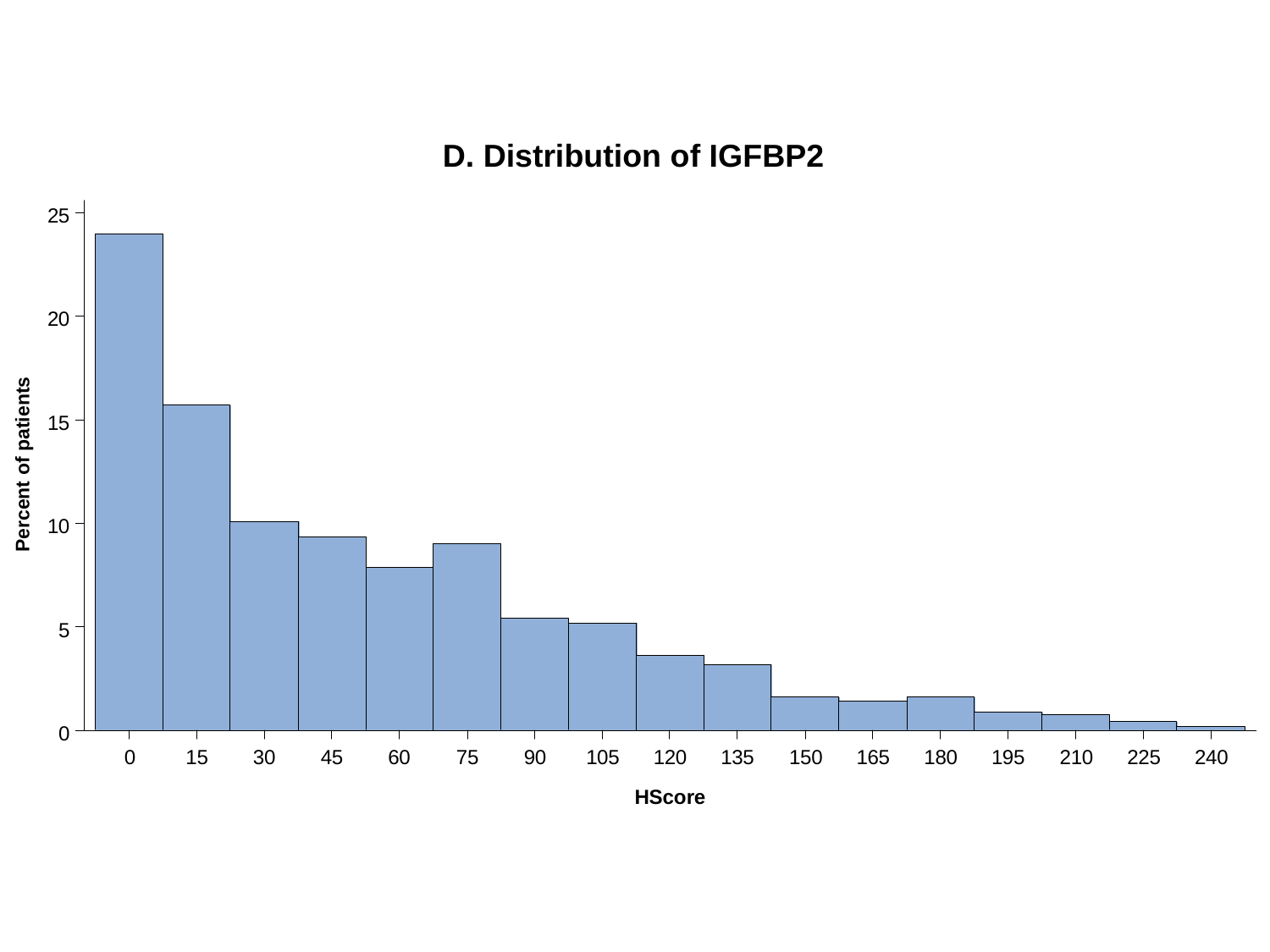

D. Distribution of IGFBP2
Percent of patients

## Slide 5
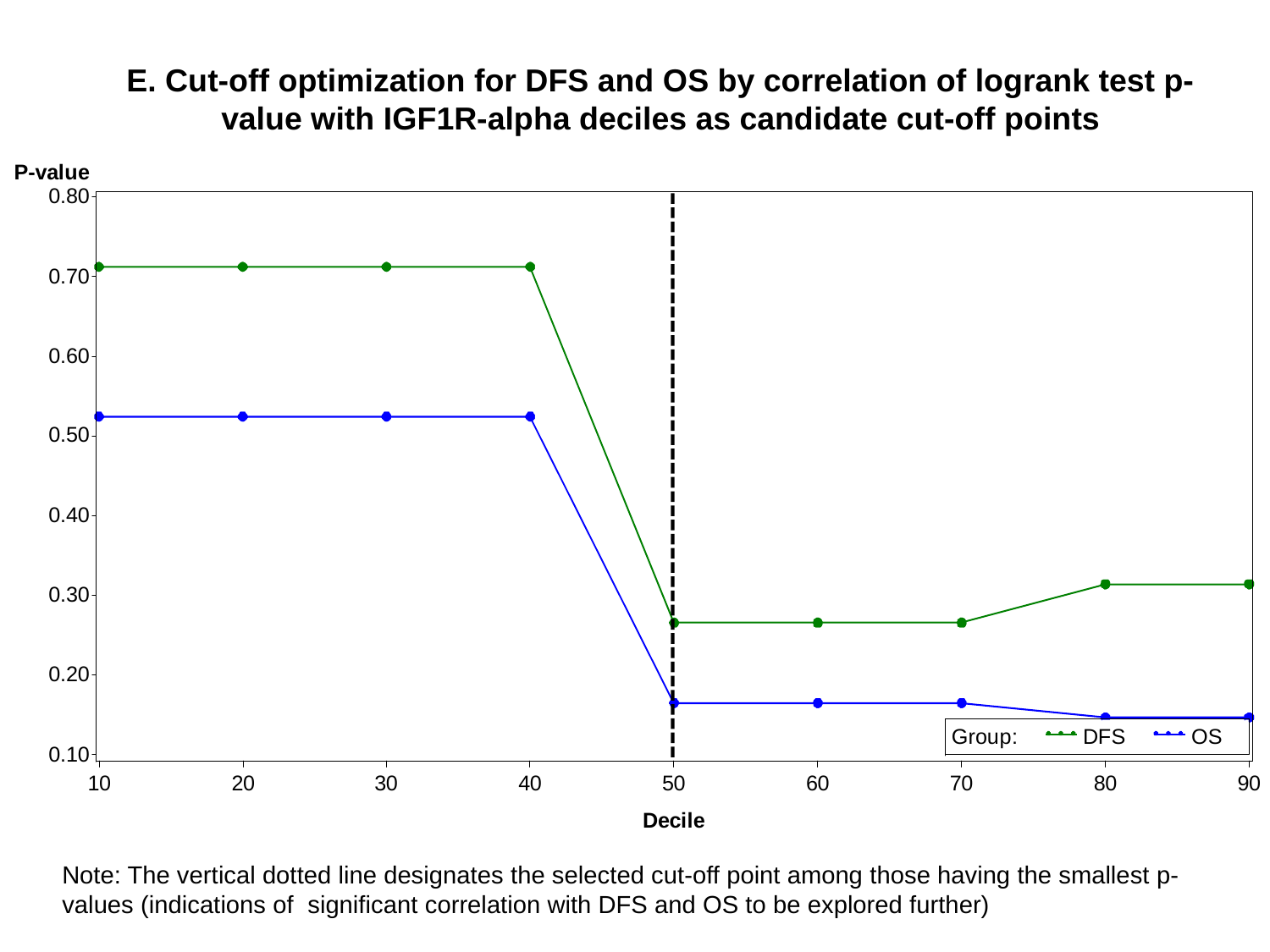

# E. Cut-off optimization for DFS and OS by correlation of logrank test p-value with IGF1R-alpha deciles as candidate cut-off points
Note: The vertical dotted line designates the selected cut-off point among those having the smallest p-values (indications of significant correlation with DFS and OS to be explored further)

## Slide 6
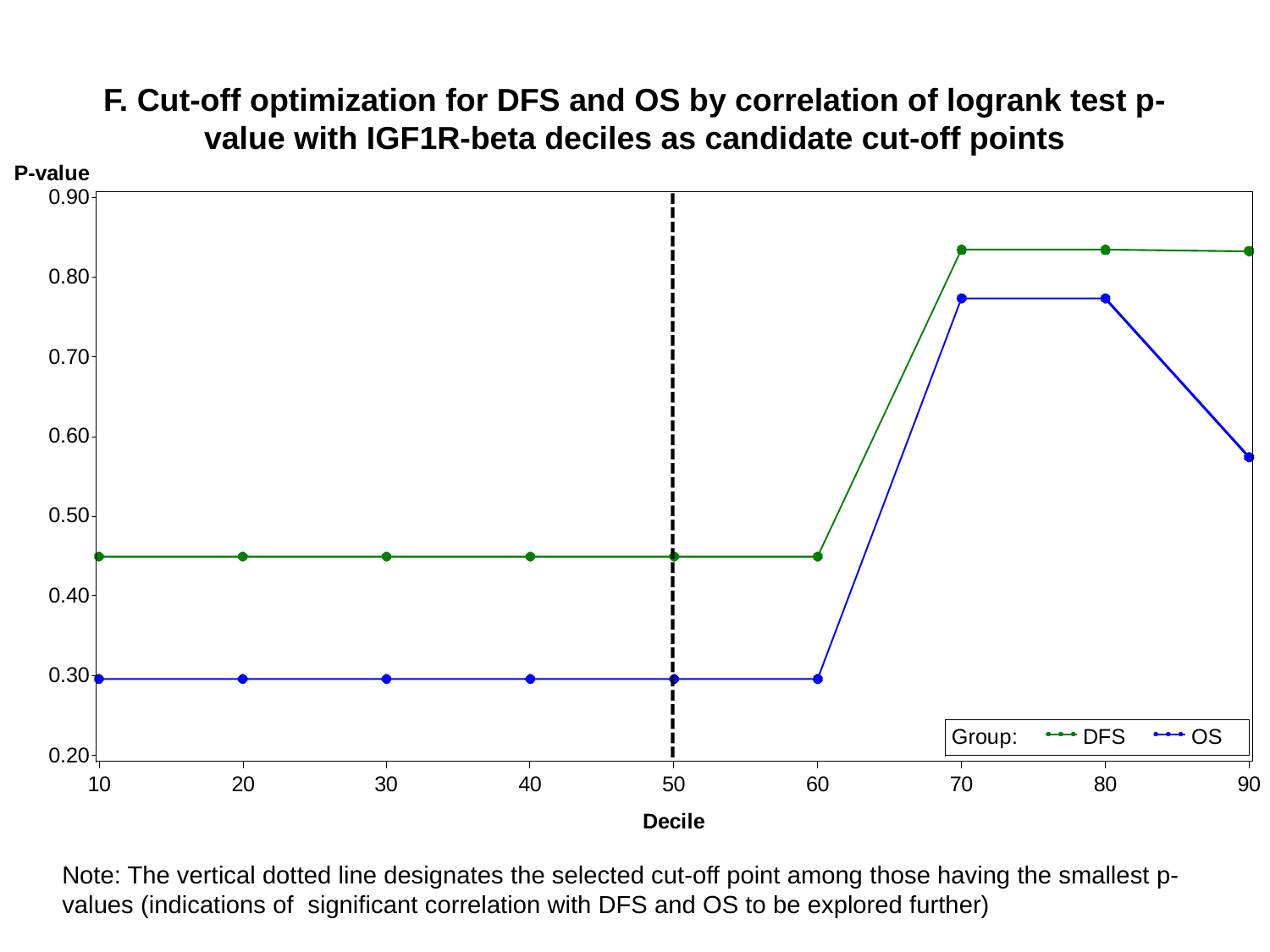

# F. Cut-off optimization for DFS and OS by correlation of logrank test p-value with IGF1R-beta deciles as candidate cut-off points
Note: The vertical dotted line designates the selected cut-off point among those having the smallest p-values (indications of significant correlation with DFS and OS to be explored further)

## Slide 7
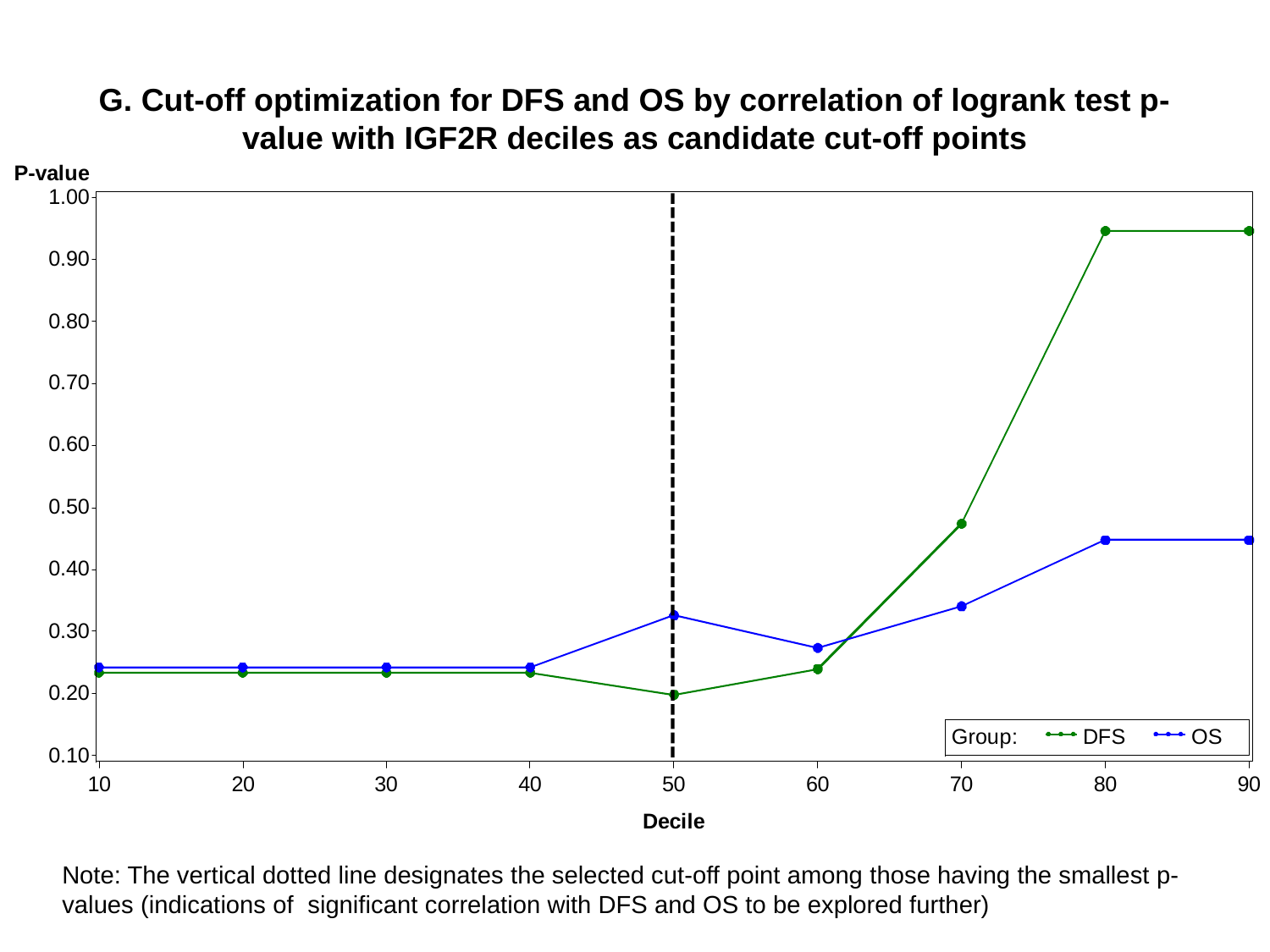

# G. Cut-off optimization for DFS and OS by correlation of logrank test p-value with IGF2R deciles as candidate cut-off points
Note: The vertical dotted line designates the selected cut-off point among those having the smallest p-values (indications of significant correlation with DFS and OS to be explored further)

## Slide 8
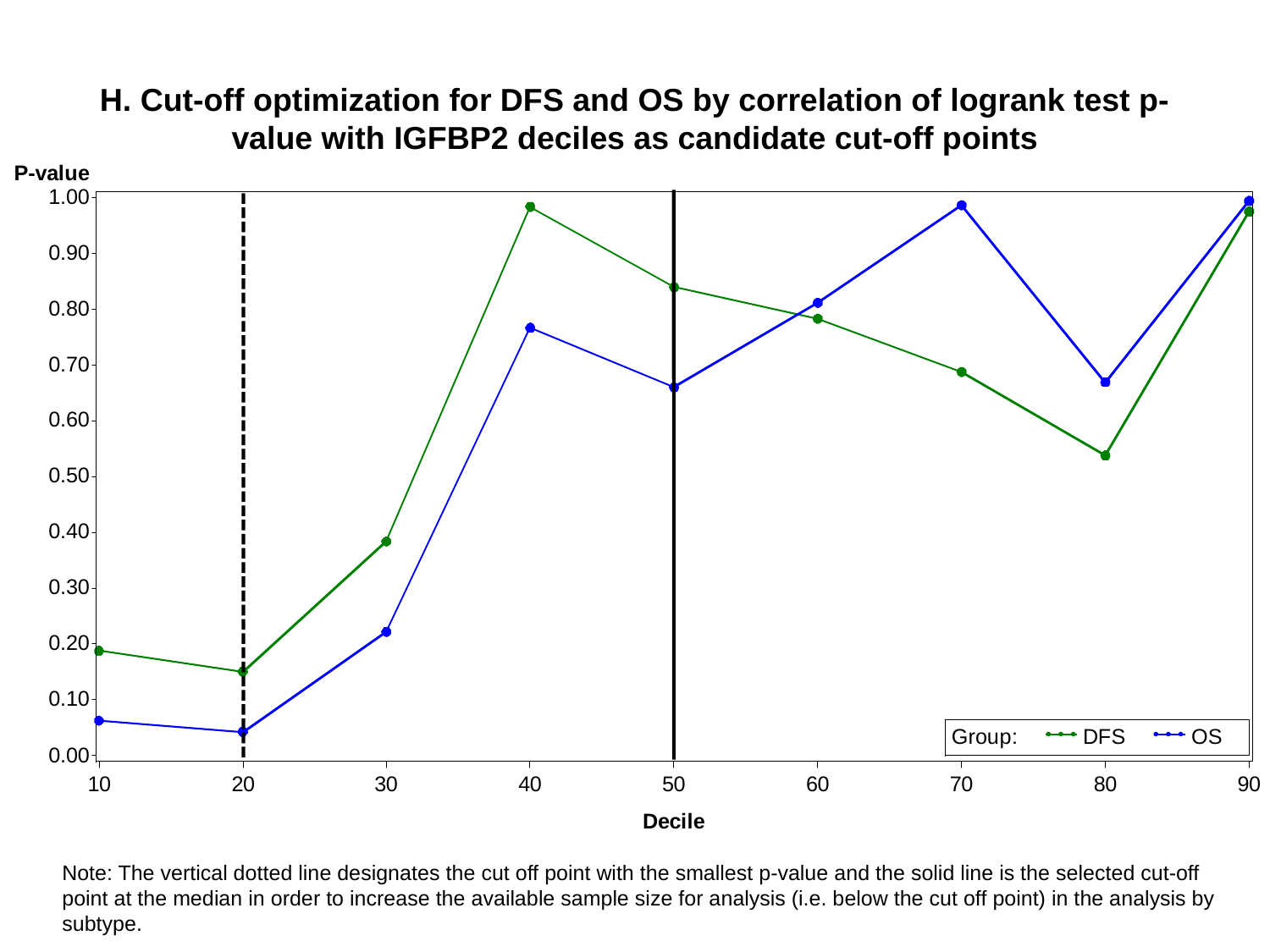

# H. Cut-off optimization for DFS and OS by correlation of logrank test p-value with IGFBP2 deciles as candidate cut-off points
Note: The vertical dotted line designates the cut off point with the smallest p-value and the solid line is the selected cut-off point at the median in order to increase the available sample size for analysis (i.e. below the cut off point) in the analysis by subtype.
