# Supplementary material for: Differential Expression of the Insulin-Like Growth Factor Receptor among Early Breast Cancer Subtypes
Source: PLoS One. 2014 Mar 17;9(3):e91407. doi: 10.1371/journal.pone.0091407 (PMC3956672; doi:10.1371/journal.pone.0091407)
Supplement: Figure S2 — Computed corresponding Hazard Ratios (HRs) for each replication and clinical endpoint (DFS/OS), for the whole study population (A–D) and the Luminal A and B patient cohort (E–H). (PPT) [file pone.0091407.s002.ppt]

## Slide 1
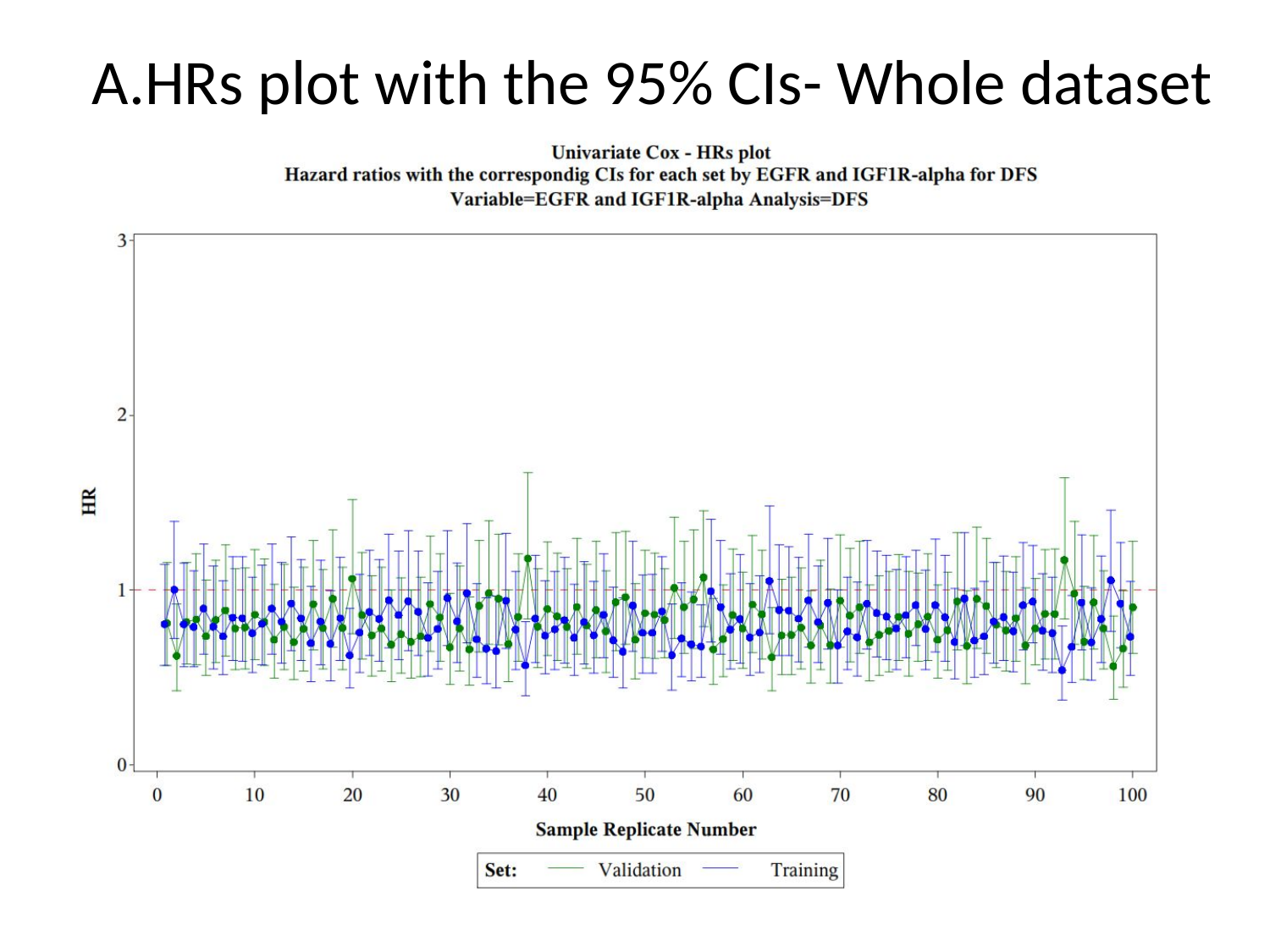

# A.HRs plot with the 95% CIs- Whole dataset

## Slide 2
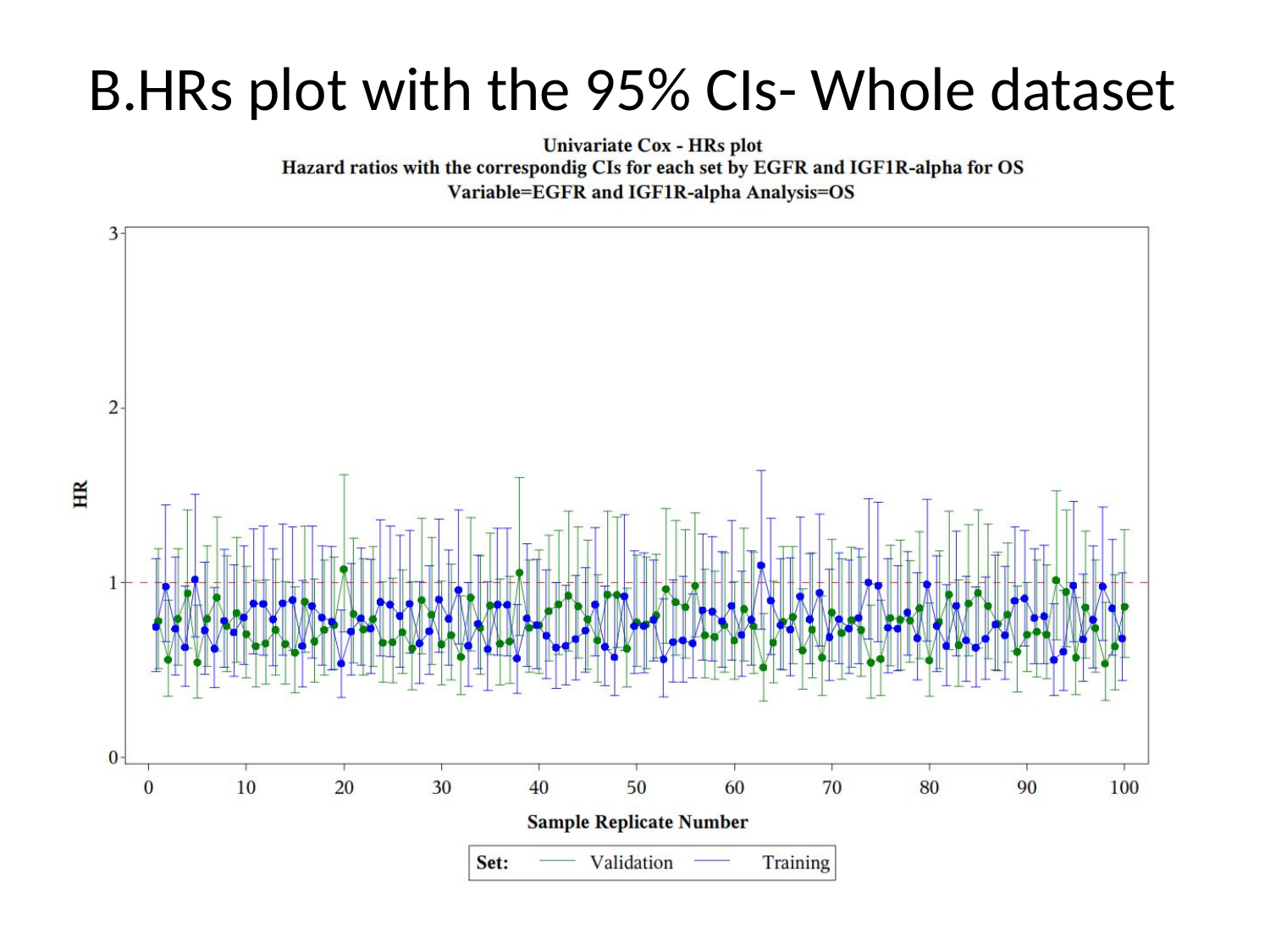

B.HRs plot with the 95% CIs- Whole dataset

## Slide 3
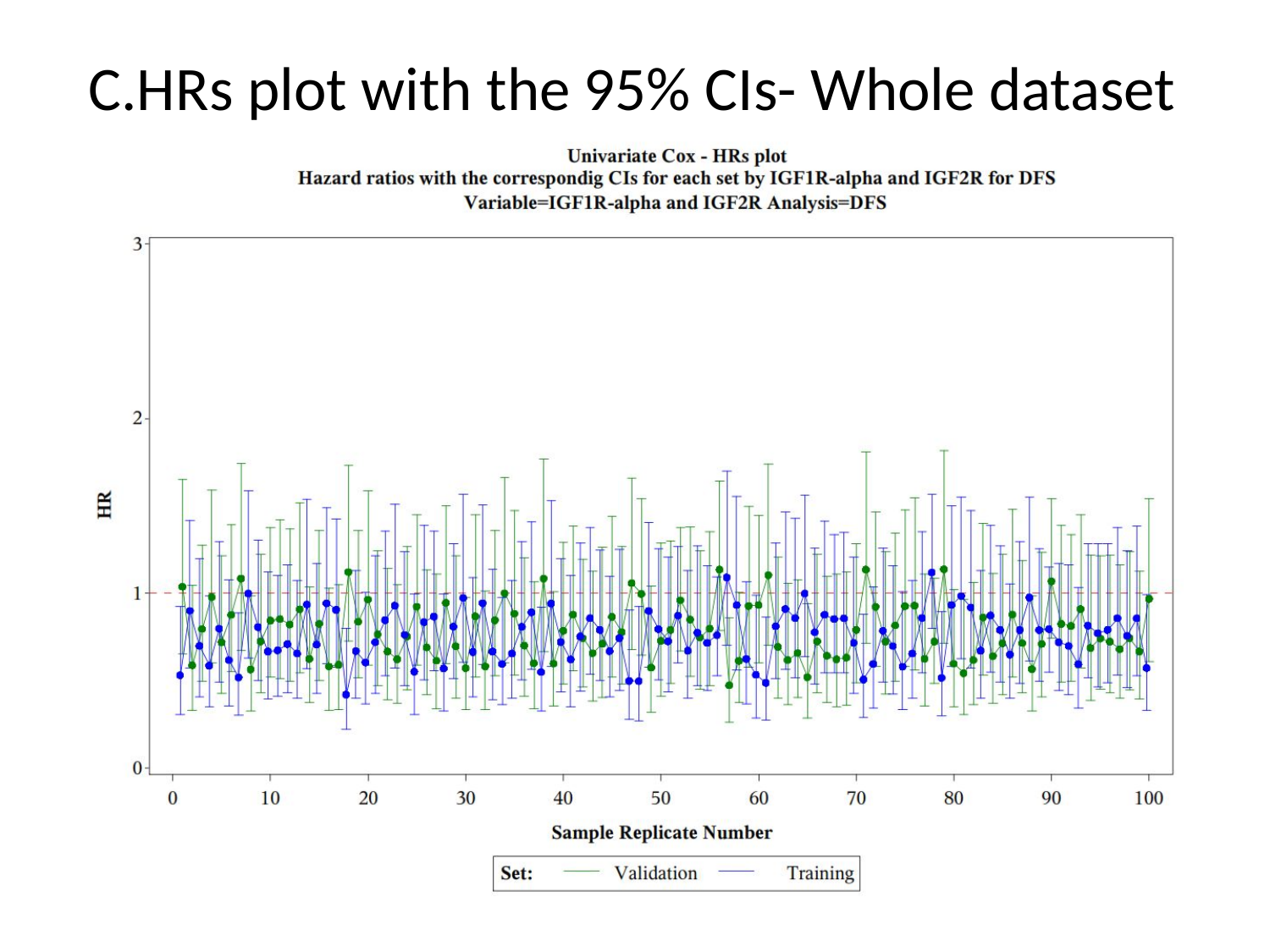

C.HRs plot with the 95% CIs- Whole dataset

## Slide 4
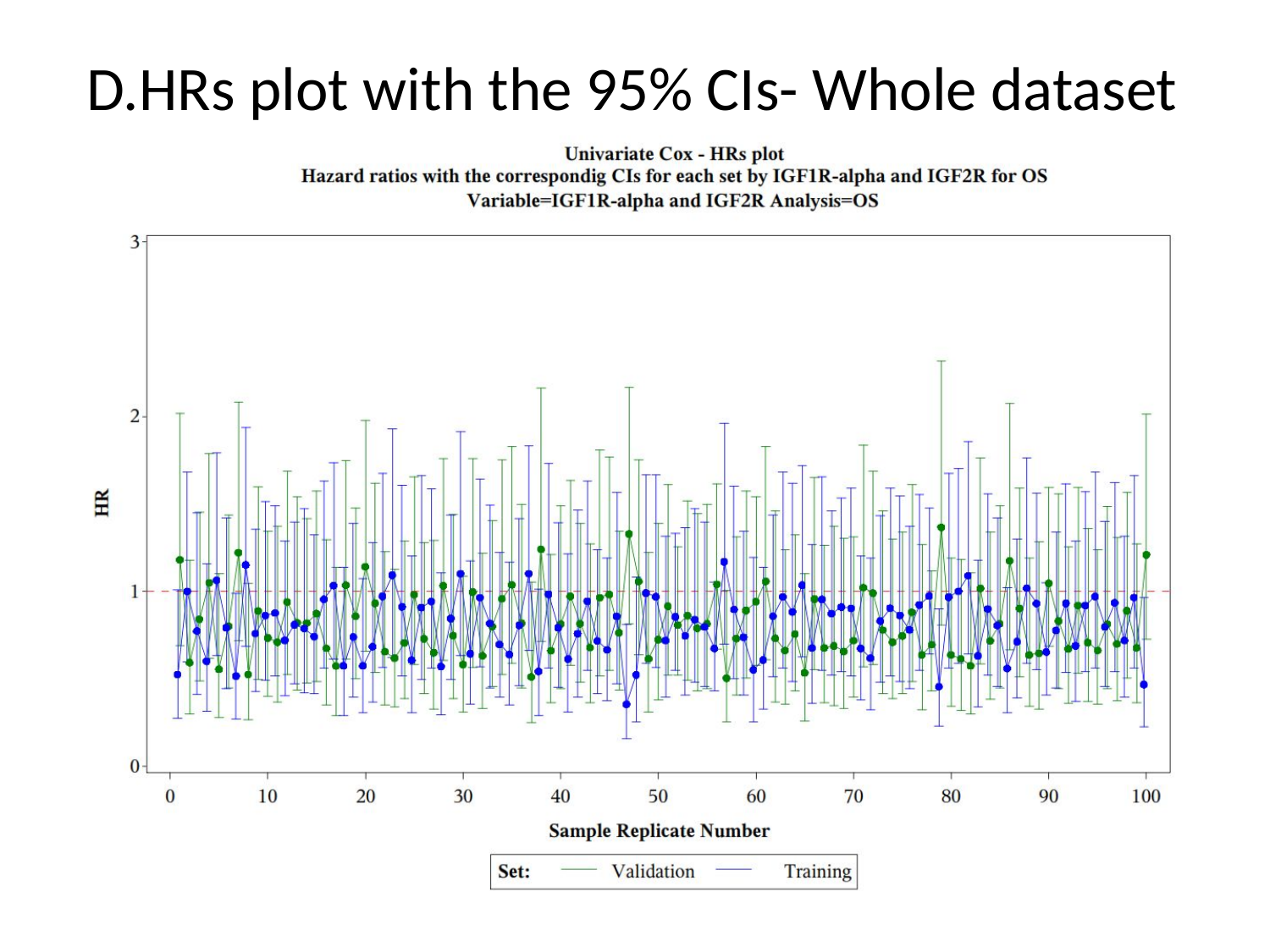

D.HRs plot with the 95% CIs- Whole dataset

## Slide 5
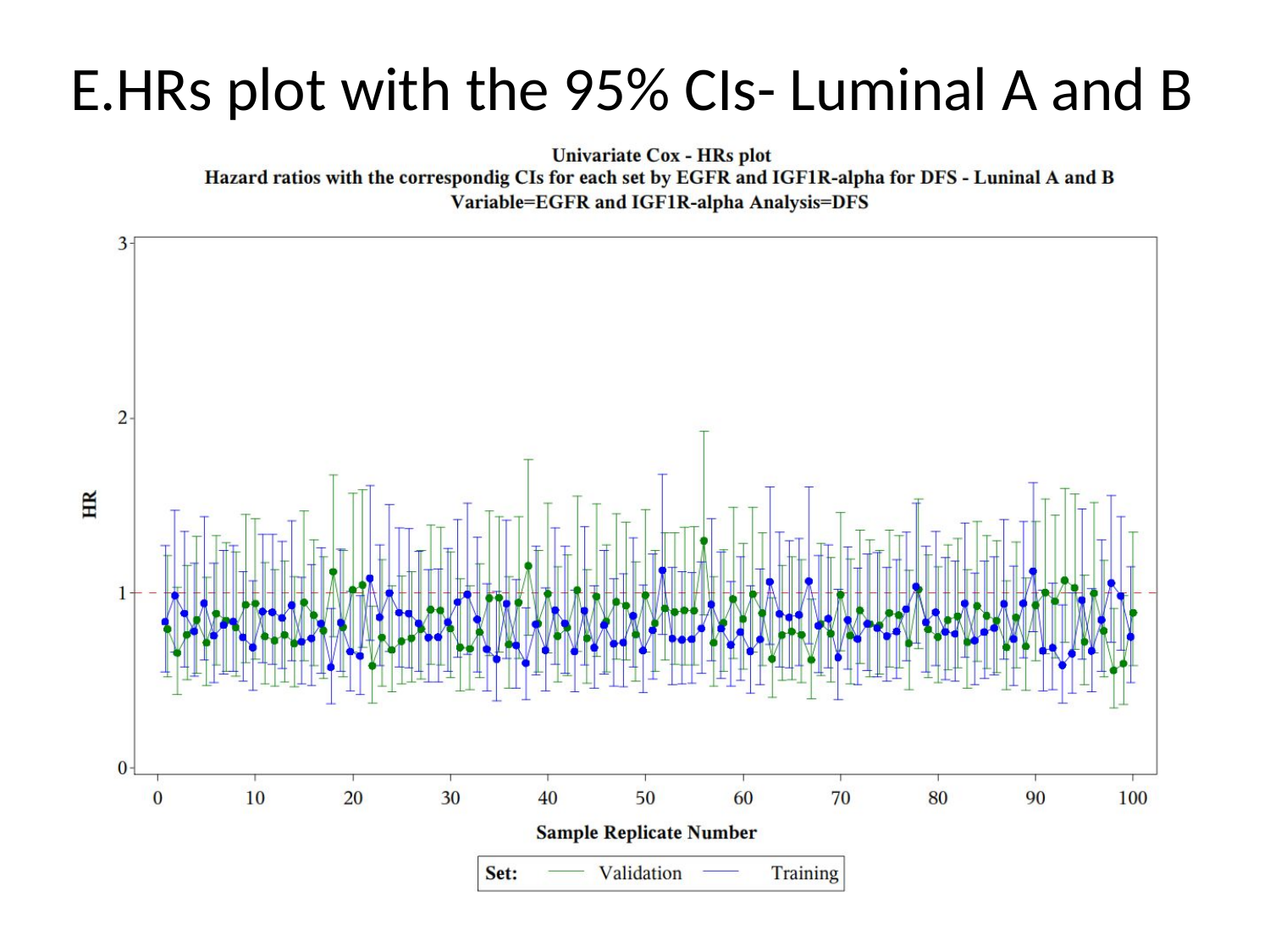

E.HRs plot with the 95% CIs- Luminal A and B

## Slide 6
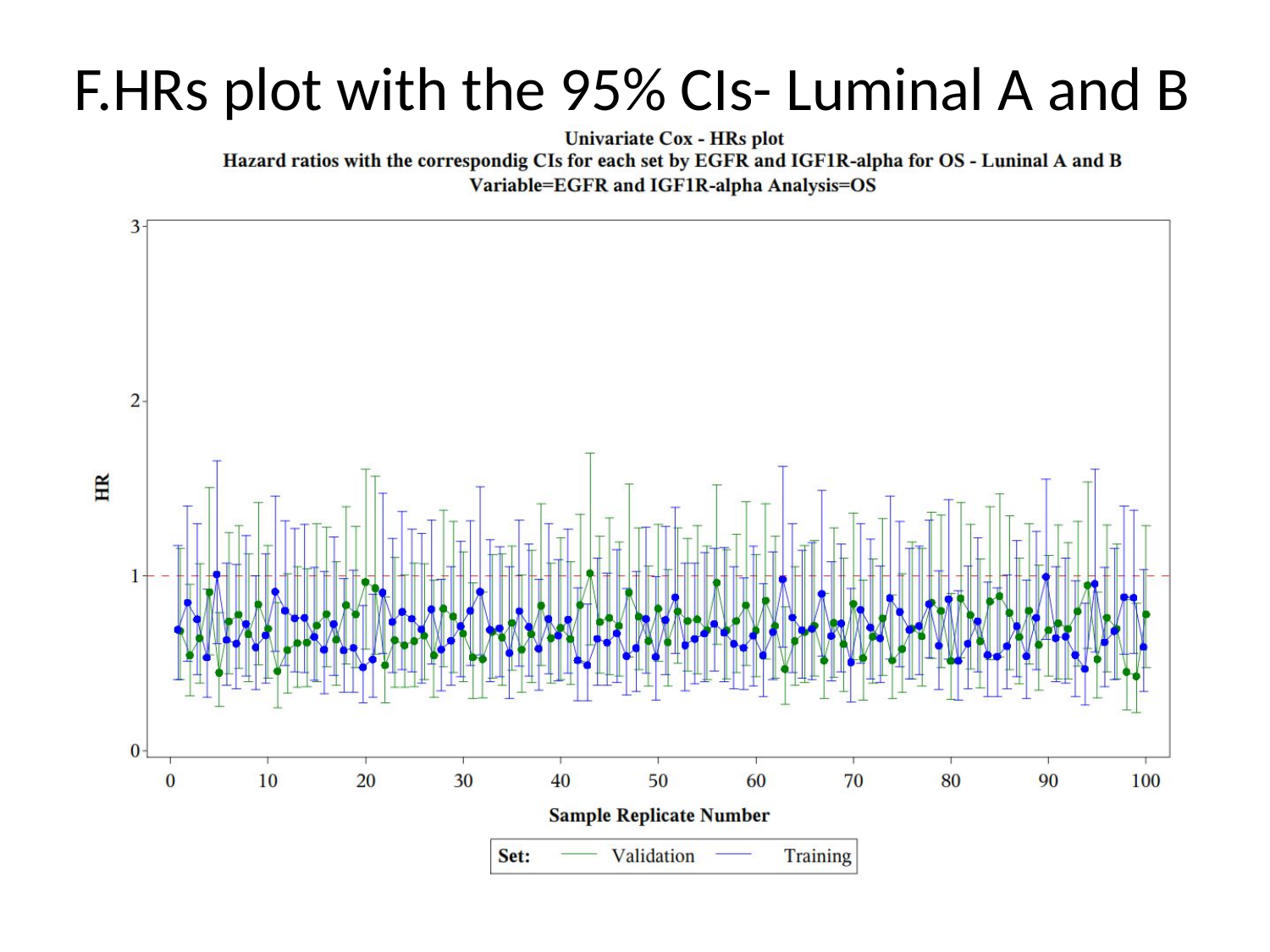

F.HRs plot with the 95% CIs- Luminal A and B

## Slide 7
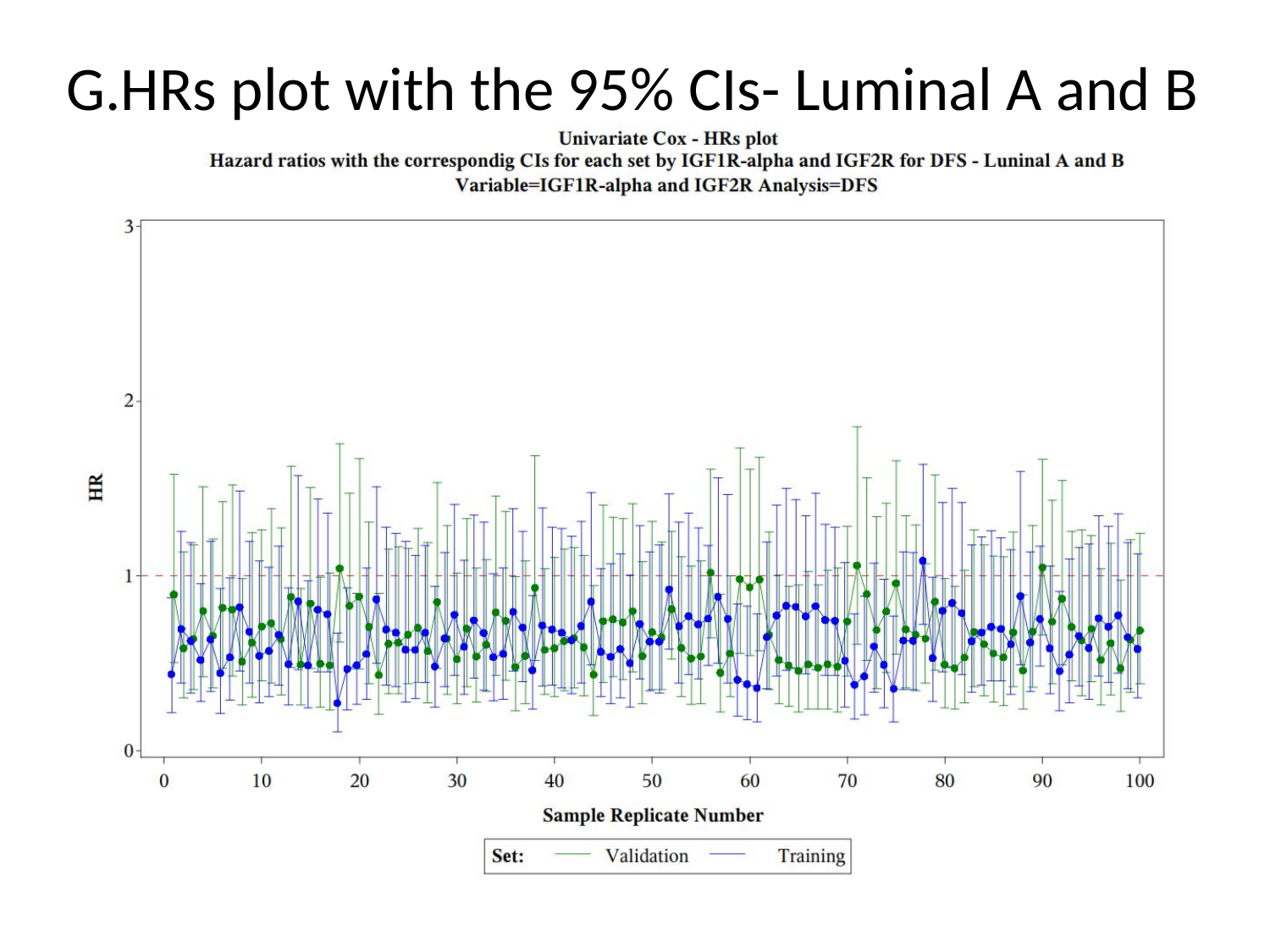

G.HRs plot with the 95% CIs- Luminal A and B

## Slide 8
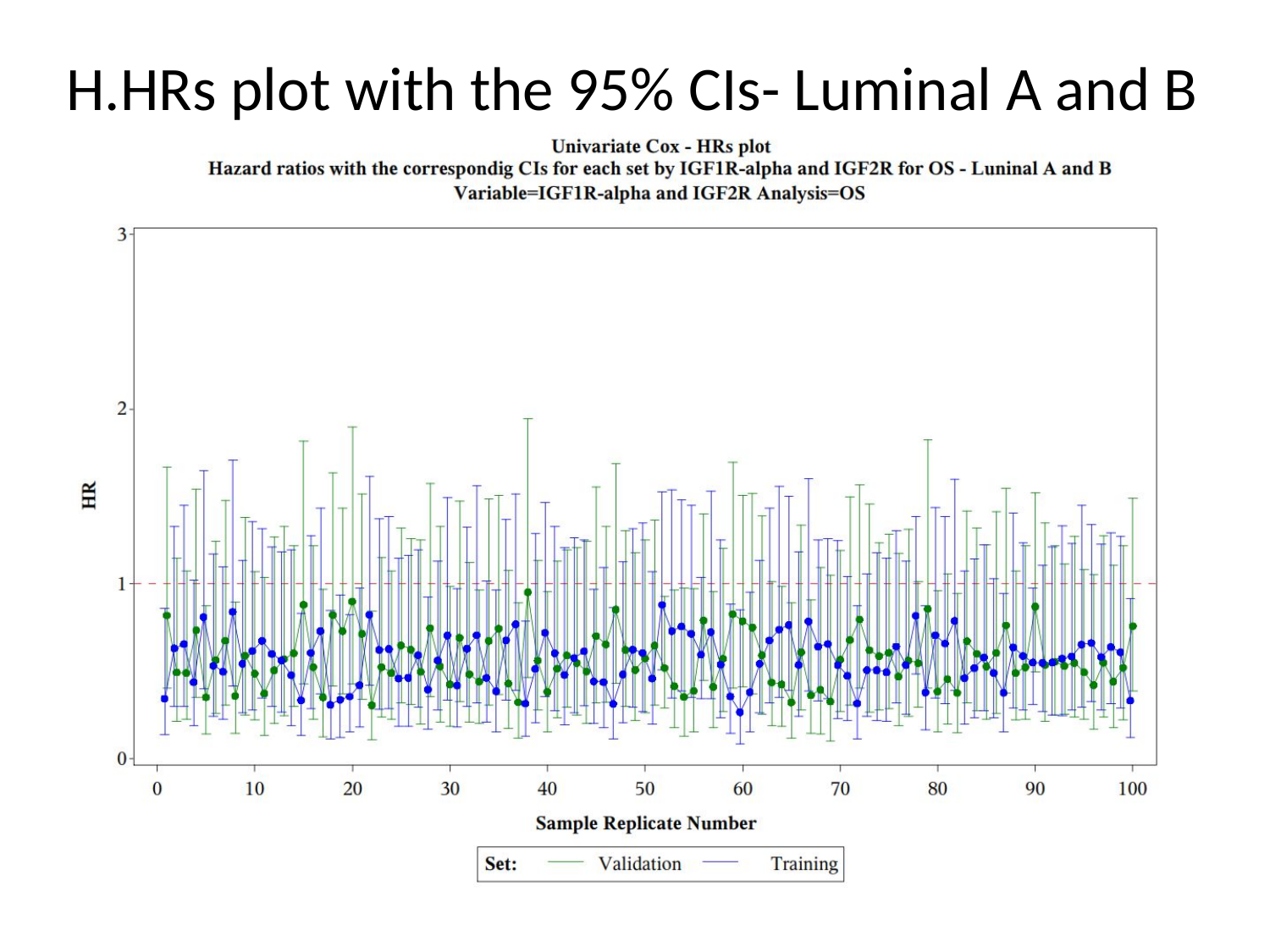

H.HRs plot with the 95% CIs- Luminal A and B
