# Supplementary material for: Differential Expression of the Insulin-Like Growth Factor Receptor among Early Breast Cancer Subtypes
Source: PLoS One. 2014 Mar 17;9(3):e91407. doi: 10.1371/journal.pone.0091407 (PMC3956672; doi:10.1371/journal.pone.0091407)
Supplement: Table S2 — Mean and median differences in Hazard Ratios (HR) of the IGF1R/IGF2R and IGF1R/EGFR clusters in the 100 splits of the training and validation set in the whole study population (A) and in Luminal A and B patients (B). (PPT) [file pone.0091407.s004.ppt]

## Slide 1
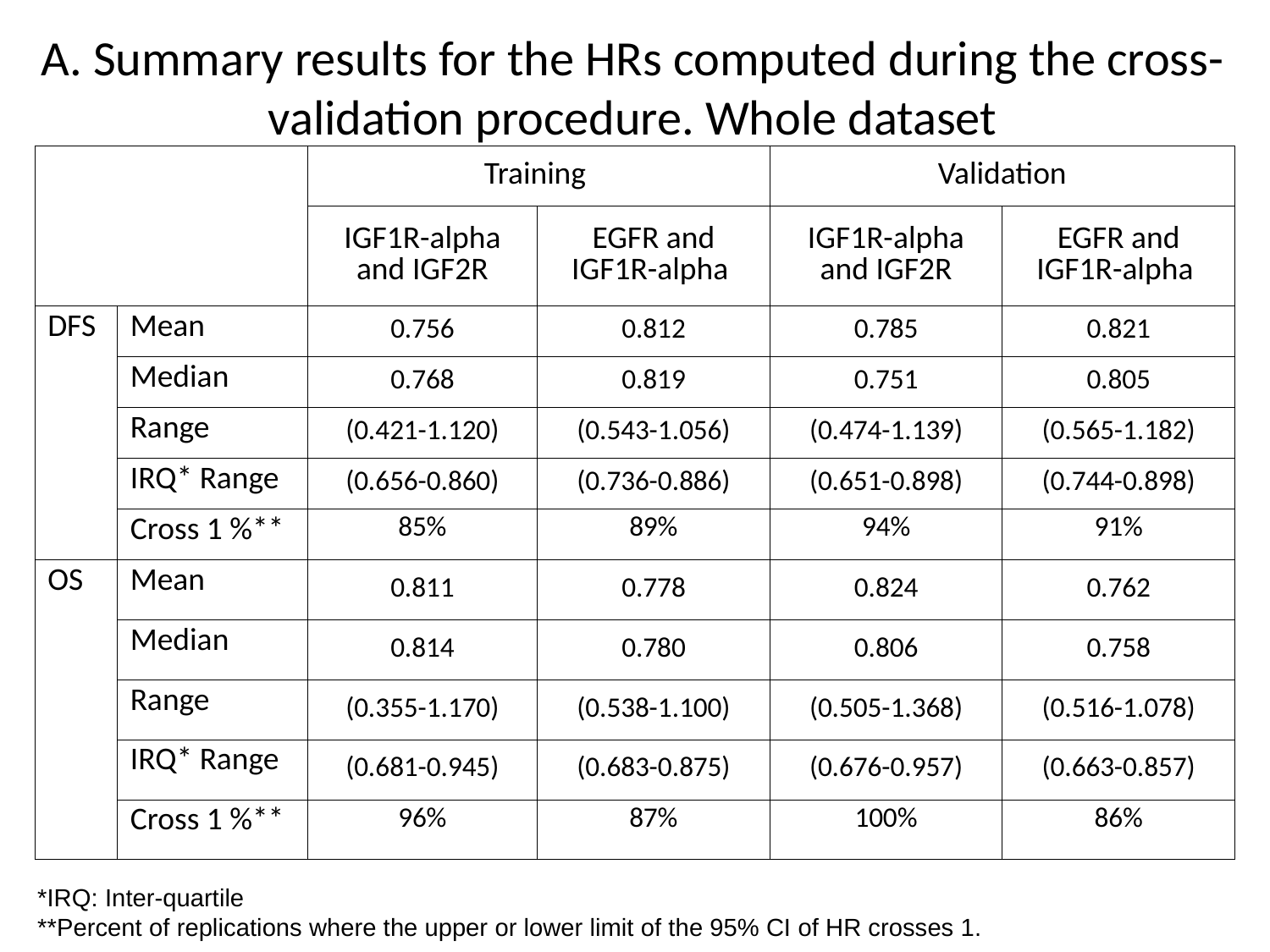

A. Summary results for the HRs computed during the cross-validation procedure. Whole dataset
| | | Training | | Validation | |
| --- | --- | --- | --- | --- | --- |
| | | IGF1R-alpha and IGF2R | EGFR and IGF1R-alpha | IGF1R-alpha and IGF2R | EGFR and IGF1R-alpha |
| DFS | Mean | 0.756 | 0.812 | 0.785 | 0.821 |
| | Median | 0.768 | 0.819 | 0.751 | 0.805 |
| | Range | (0.421-1.120) | (0.543-1.056) | (0.474-1.139) | (0.565-1.182) |
| | IRQ\* Range | (0.656-0.860) | (0.736-0.886) | (0.651-0.898) | (0.744-0.898) |
| | Cross 1 %\*\* | 85% | 89% | 94% | 91% |
| OS | Mean | 0.811 | 0.778 | 0.824 | 0.762 |
| | Median | 0.814 | 0.780 | 0.806 | 0.758 |
| | Range | (0.355-1.170) | (0.538-1.100) | (0.505-1.368) | (0.516-1.078) |
| | IRQ\* Range | (0.681-0.945) | (0.683-0.875) | (0.676-0.957) | (0.663-0.857) |
| | Cross 1 %\*\* | 96% | 87% | 100% | 86% |
*IRQ: Inter-quartile
**Percent of replications where the upper or lower limit of the 95% CI of HR crosses 1.

## Slide 2
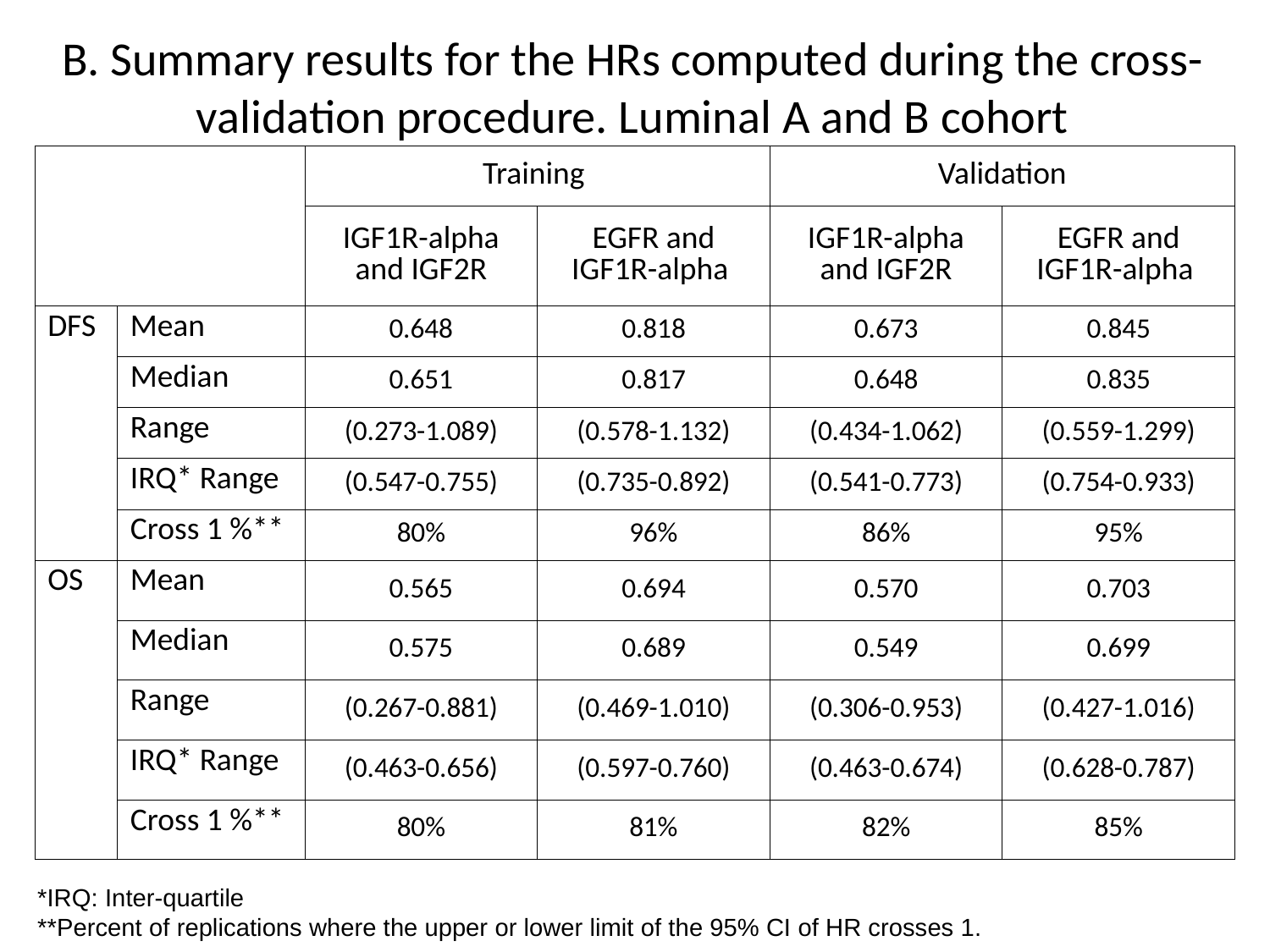

B. Summary results for the HRs computed during the cross-validation procedure. Luminal A and B cohort
| | | Training | | Validation | |
| --- | --- | --- | --- | --- | --- |
| | | IGF1R-alpha and IGF2R | EGFR and IGF1R-alpha | IGF1R-alpha and IGF2R | EGFR and IGF1R-alpha |
| DFS | Mean | 0.648 | 0.818 | 0.673 | 0.845 |
| | Median | 0.651 | 0.817 | 0.648 | 0.835 |
| | Range | (0.273-1.089) | (0.578-1.132) | (0.434-1.062) | (0.559-1.299) |
| | IRQ\* Range | (0.547-0.755) | (0.735-0.892) | (0.541-0.773) | (0.754-0.933) |
| | Cross 1 %\*\* | 80% | 96% | 86% | 95% |
| OS | Mean | 0.565 | 0.694 | 0.570 | 0.703 |
| | Median | 0.575 | 0.689 | 0.549 | 0.699 |
| | Range | (0.267-0.881) | (0.469-1.010) | (0.306-0.953) | (0.427-1.016) |
| | IRQ\* Range | (0.463-0.656) | (0.597-0.760) | (0.463-0.674) | (0.628-0.787) |
| | Cross 1 %\*\* | 80% | 81% | 82% | 85% |
*IRQ: Inter-quartile
**Percent of replications where the upper or lower limit of the 95% CI of HR crosses 1.
