# Supplementary material for: Differential Expression of the Insulin-Like Growth Factor Receptor among Early Breast Cancer Subtypes
Source: PLoS One. 2014 Mar 17;9(3):e91407. doi: 10.1371/journal.pone.0091407 (PMC3956672; doi:10.1371/journal.pone.0091407)
Supplement: Table S3 — Bivariate associations between immunohistochemical expression of biomarkers. (PPT) [file pone.0091407.s005.ppt]

## Slide 1
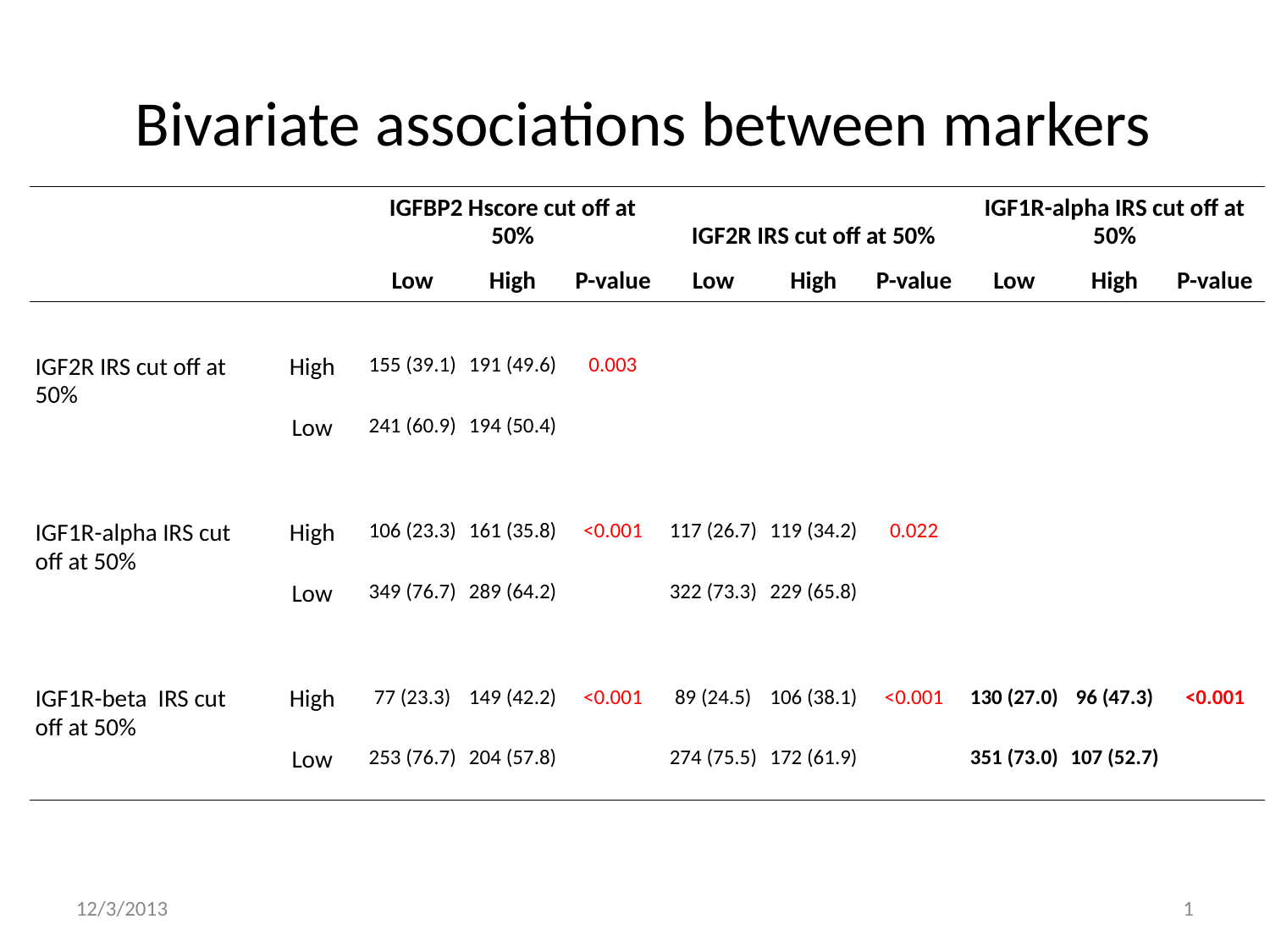

# Bivariate associations between markers
| | | IGFBP2 Hscore cut off at 50% | | | IGF2R IRS cut off at 50% | | | IGF1R-alpha IRS cut off at 50% | | |
| --- | --- | --- | --- | --- | --- | --- | --- | --- | --- | --- |
| | | Low | High | P-value | Low | High | P-value | Low | High | P-value |
| | | | | | | | | | | |
| IGF2R IRS cut off at 50% | High | 155 (39.1) | 191 (49.6) | 0.003 | | | | | | |
| | Low | 241 (60.9) | 194 (50.4) | | | | | | | |
| | | | | | | | | | | |
| IGF1R-alpha IRS cut off at 50% | High | 106 (23.3) | 161 (35.8) | <0.001 | 117 (26.7) | 119 (34.2) | 0.022 | | | |
| | Low | 349 (76.7) | 289 (64.2) | | 322 (73.3) | 229 (65.8) | | | | |
| | | | | | | | | | | |
| IGF1R-beta IRS cut off at 50% | High | 77 (23.3) | 149 (42.2) | <0.001 | 89 (24.5) | 106 (38.1) | <0.001 | 130 (27.0) | 96 (47.3) | <0.001 |
| | Low | 253 (76.7) | 204 (57.8) | | 274 (75.5) | 172 (61.9) | | 351 (73.0) | 107 (52.7) | |
12/3/2013
<number>
